# Resting-state fMRI functional connectivity of the left temporal parietal junction is associated with visual temporal order threshold

**DOI:** 10.1038/s41598-022-20309-1

**Published:** 2022-09-24

**Authors:** Monika Lewandowska, Jan Nikadon, Tomasz Wolak, Krzysztof Tołpa, Tomasz Piotrowski, Mateusz Chojnowski, Joanna Dreszer

**Affiliations:** 1 Institute of Psychology, Faculty of Philosophy and Social Sciences, Nicolaus Copernicus University in Torun, 39 Gagarina Street, 87-100 Toruń, Poland; 2 Center for Research on Social Relations, SWPS University of Social Sciences and Humanities, 19/31 Chodakowska Street, 03-815 Warsaw, Poland; 3 Bioimaging Research Center, World Hearing Center, Institute of Physiology and Pathology of Hearing, Warsaw/Kajetany, Poland; 4 Centre for Modern Interdisciplinary Technologies, Nicolaus Copernicus University in Torun, 4 Wilenska Street, 87-100 Toruń, Poland; 5 Department of Informatics, Nicolaus Copernicus University in Torun, 5 Grudziadzka Street, 87-100 Toruń, Poland

**Keywords:** Cognitive neuroscience, Attention, Perception, Sensory processing

## Abstract

The study aimed to determine the relationship between the millisecond timing, measured by visual temporal order threshold (TOT), i.e. a minimum gap between two successive stimuli necessary to judge a before-after relation, and resting-state fMRI functional connectivity (rsFC). We assume that the TOT reflects a relatively stable feature of local internal state networks and is associated with rsFC of the temporal parietal junction (TPJ). Sixty five healthy young adults underwent the visual TOT, fluid intelligence (G_f_) and an eyes-open resting-state fMRI examination. After controlling for the influence of gender, the higher the TOT, the stronger was the left TPJ’s rsFC with the left postcentral and the right precentral gyri, bilateral putamen and the right supplementary motor area. When the effects of G_f_ and TOT × G_f_ interaction were additionally controlled, the TOT—left TPJ’s rsFC relationship survived for almost all above regions with the exception of the left and right putamen. This is the first study demonstrating that visual TOT is associated with rsFC between the areas involved both in sub-second timing and motor control. Current outcomes indicate that the local neural networks are prepared to process brief, rapidly presented, consecutive events, even in the absence of such stimulation.

## Introduction

Cognitive processes can be better described and understood when their temporal frames will be determined. Temporal experiences such as simultaneity, successiveness or subjective present, are thought to be organized hierarchically^1,2^ and one of the most basic time experience is the identification of temporal order of events reaching the brain within tens of milliseconds. It is usually measured by the Temporal Order Threshold (TOT), i.e. the minimal inter-stimulus interval (ISI) between two consecutive stimuli necessary to report their before—after relation with a high (usually > 75%) accuracy^3^. The TOT values typically lie in the range from 20 to 60 ms., independently of the sensory modality^4,5^, suggesting a central mechanism of temporal order judgment (TOJ). Since these threshold values were also found to be affected by the physical features of the stimuli employed^6,7^, TOJ could be also governed by the modality- specific, sensory-automatic timing system^8^. The TOT is elevated in fluent aphasia^9^, Specific Language Impairment^10^, dyslexia^11^, Attention-Deficit/Hyperactivity Disorder^12^ or normal aging^13^ and is associated with fluid intelligence (G_f_)^14–18^, attention^13,18^ and working memory^3,18,19^. Any activity within the millisecond time scale (e.g. rapid movements, coordination of muscles during motor actions) is supposed to be automatic, beyond cognitive control^20^. Recently, Chassignolle et al.^21^ demonstrated that even when the temporal order of two visual stimuli are not perceived consciously (because a gap between their onsets was < 20 ms.), it still could be processed. When an ISI is extremely low the before—after relation still could be determined, but specific perceptual strategies are then involved^22–24^.

The neural underpinnings of TOJs are known quite well as revealed by the fMRI^25–29^, EEG^30–34^, TMS^35,36^ or lesion^9,37,38^ studies. The most consistent finding here is the involvement of the temporal parietal junction (TPJ) which is the region surrounding the posterior end of the Sylvian fissure that covers the inferior parietal lobule (the angular and supramarginal gyri) as well as posterior extents of the superior temporal gyrus and sulcus^39^. There is a little controversy whether the left or right TPJ is more recruited by TOJs. TOT was found to be elevated after either the left^9^ or right^38^ hemisphere damage. Bilateral activations of this region were observed in the fMRI paradigm where the participants reported the order of two brief colorful rectangles appearing in the opposite corners of the screen (in contrast to the shape judgments)^27^. The modification of this procedure ensured that both TOJ and the control task required attending to the stimulus onsets and in this case only the left TPJ was recruited. The shifting of the EEG signal source from bilateral to the left TPJ along with the TOJ improvement^30^ as well as stronger global power spectra of pre-stimulus beta activity (18–23 Hz) was observed when the TOJs were more accurate compared to the inaccurate condition^32^. Estimated source of these high-frequency oscillations was located in bilateral TPJ during the whole task but with the left-hemispheric dominance whenever the TOJs were more correct. The relationship between the TOJ task accuracy and activity of the left temporal cortex within the 39–77 ms. post-stimulus period was also found^31^. In the latter study the functional connectivity between the right and left TPJ was observed but only during the inaccurate TOJ condition, suggesting that the interactions between these two homotopic regions significantly affect the temporal order judgments. There is also evidence demonstrating that the multisensory TOJs involve more global neural networks, not restricted to the TPJ. The audiovisual TOJ task is thought to activate mainly the frontoparietal network subserving attention and cognitive control^25,26^. When the before—after relation of two tactile stimuli was determined (in contrast to judging the number of points of tactile stimulation)^29^ bilateral activations in the premotor cortex, middle frontal gyrus, inferior parietal cortex, supramarginal gyrus and the posterior part of the superior and middle temporal gyri were found. The regions, found in the latter study, are considered as being motion-sensitive and/or involved in localizing incoming events in space.

In the current work we sought to determine whether the millisecond timing, measured by means of visual TOT, is associated with the resting-state fMRI functional connectivity (rsFC) in young, healthy individuals. Neural correlates of TOJ have been investigated predominantly with the use of active tasks but it seems reasonable to believe that the ability to identify the temporal order of rapidly presented events is also reflected in spontaneous brain activity. Back in time, Pinneo^40^ suggested that baseline, task-independent “tonic” rather than “phasic”, task-induced activity, is crucial for the neural system functioning. Any variations in the resting-state low-frequency fluctuations may represent a logistical basis for the system (micro)states within which information, distributed in the spatiotemporal domain, can be integrated^41,42^. The TOJ tasks require to organize incoming stimuli in time and space and this process appears to be ‘primordial’ or fundamental for any conscious experience^1^. According to the hypothetical model of time perception^1^, the brain constantly creates elementary processing units within which incoming information is treated as co-temporal. These "atemporal" units or states last ca. 20–60 ms. and provide the logistical basis for the identification of successive events. Consequently, we assume that the TOT, as a measure of the system’s elementary state duration, would be associated with spontaneous neural activity and this relationship reflects the brain’s readiness to encounter stimuli appearing in rapid succession. Since there is a high correspondence of the brain’s functional organization during activation and resting state^43,44^, we will search for correlations between the TOT values and rsFC of the TPJ, typically involved in various TOJ tasks^30–32^.

Our previous study has demonstrated a decrease or increase of an ISI between two brief tones, the order of which was being assessed, accompanied by different fMRI activation patterns^28^. These outcomes could be explained by a greater amount of mental effort allocated to identify the sounds separated by an ISI much below the threshold value (10 ms.). Therefore, we assume that the temporal thresholds will be associated with the interactions between the TPJs and other brain areas involved in attentional processes and/or specific perceptual strategies used to cope with rapidly presented consecutive stimuli (e.g. two visual objects at different positions may give the impression of apparent motion and then the involvement of timing processes is no longer necessary).

We also hypothesize that since the temporal order perception is associated with intellectual abilities^17,18,45^ and discrimination of extremely brief intervals represents a sensitive G_f_ indicator^17,45,46^, the relationship between the TOT and the TPJ’s resting-state functional couplings will be influenced by fluid intelligence level. Furthermore, we may assume that switching to a holistic strategy while making TOJs (integration the single visual stimuli into one percept) can be done more easily by the individuals with higher intellectual skills^18^. Consequently, if they readily use this perceptual mechanism, the TOJ task will become “nontemporal” for them and the role of TPJ may be then reduced. Therefore, the relationship between the TOT and the TPJ’s spontaneous functional connectivity may be preserved mainly in the individuals with lower G_f_ factor level who less likely use the specific perceptual strategies in the TOJ task.

## Results

### Behavioral results

**Figure 1:**
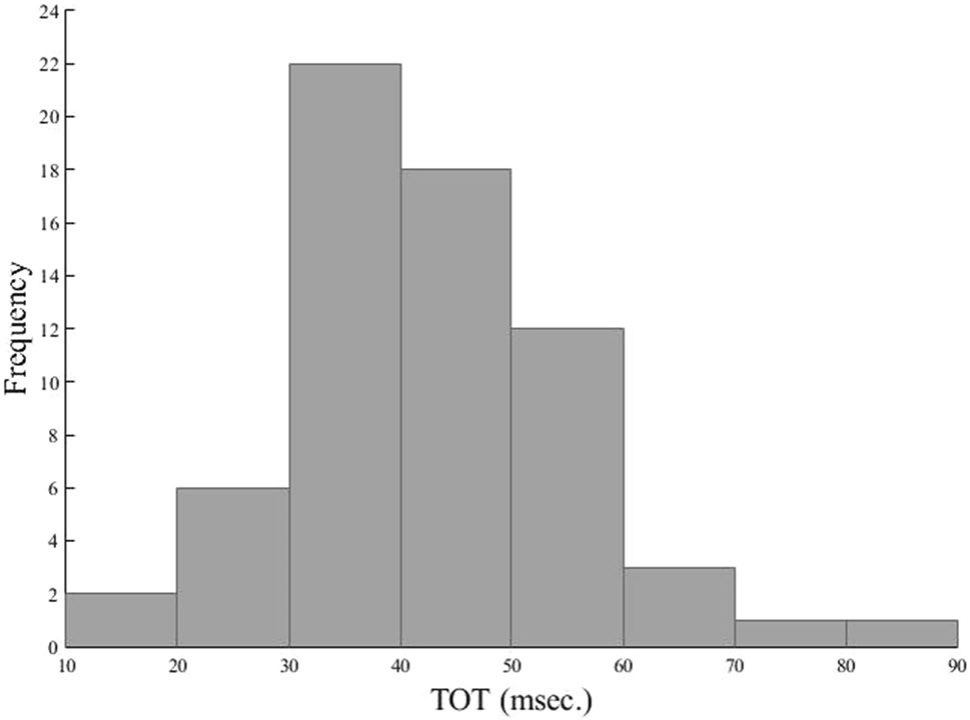
The distribution of the temporal order threshold (TOT) values across all the participants (n = 65). The mean TOT value = 42.86 ms., Standard Deviation (SD) = 12.7 ms., the median TOT value = 44 ms. and the TOT range: 18.67–86.47 ms. The skewness = 0.94 and the kurtosis = 1.65.

The distribution of the TOT values in all participants (n = 65) is shown in Fig. 1. The TOT negatively correlated with the G_f_ factor (rho = − 0.429, p < 0.001).

### Seed-Based Connectivity (SBC) results

**Figure 2:**
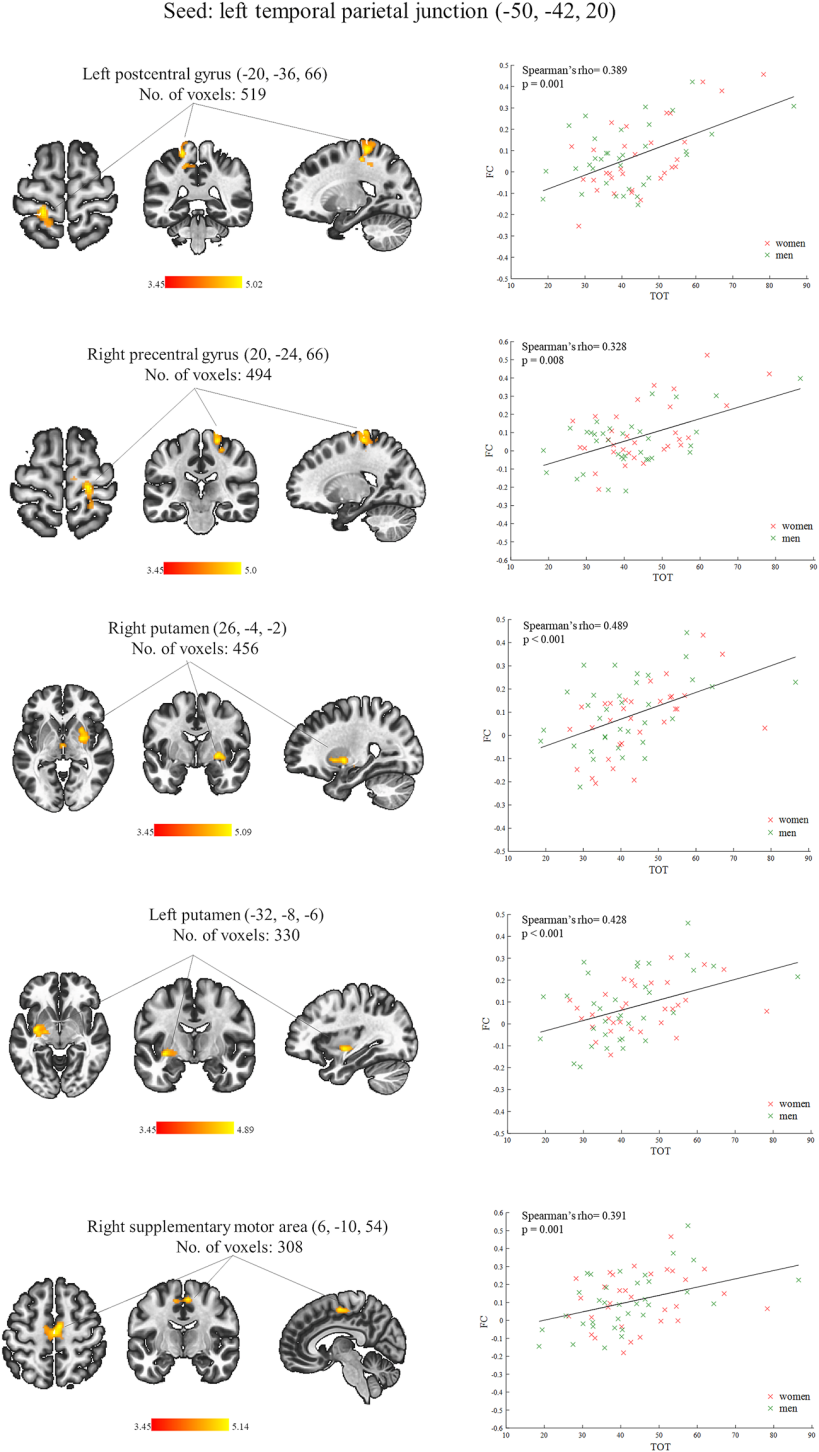
(Left) Brain regions demonstrating a significant positive association between the temporal order threshold (TOT) values and functional connectivity (FC) values seeded from the left temporal parietal junction while the gender effect was controlled. The Montreal Neurological Institute (MNI) coordinates of center position of the seed area are put right after the name of it, in the bracket. The size of the whole target region is given in the number of voxels (each voxel size: 2 × 2 × 2 mm), and the name indicates the anatomical area the majority of the voxels belonged to. The color bars represent T scores. (Right) Scatter plots of functional connectivity (FC) parameters as a function of the temporal order threshold (TOT) at the peak voxel of each target region. The FC values for women are marked in red and for men in green. The black solid line represents a best fit for linear regression on a datasets with outliers (fit robust linear regression). The values of Spearman’s rho coefficients that measure the strength of association between the TOT and FC and their significance levels (p) are placed in the left upper corner of each scatter plot.

The TOT was related to functional couplings of only one from the predefined seed regions. After controlling for the effect of gender, there were positive correlations between the TOT values and the rsFC of the left TPJ that corresponds to the left superior temporal gyrus, with the left postcentral gyrus (PostCG), right precentral gyrus (PreCG), bilateral putamen and the right supplementary motor area (SMA). These outcomes are shown in Fig. 2 and Table 1 (the effects were thresholded at the cluster-mass FDR-corrected level of p < 0.05 and Bonferroni corrected at p < 0.05). There were no significant negative correlations between the TOT and rsFC of any seed region.

**Table 1:** Resting-state functional connectivity found correlated positively with the temporal order threshold (TOT) values in all participants (n = 65) after controlling for gender.

	Seed region/MNI coordinates (x, y, z)	Main region in the target area	No of voxels	MNI coordinates (x, y, z)	p-value FDR_C_ p < 0.05 corr and Bonferroni p < 0.05 corr
1	Left temporal parietal junction (− 50, − 42, 20)	Left postcentral gyrus	519	− 20, − 36, 66	0.002102
		Right precentral gyrus	494	20, − 24, 66	0.002102
		Right putamen	456	26, − 4, − 2	0.002102
		Left putamen	330	− 32, − 8, − 6	0.003349
		Right supplementary motor area	308	6, − 10, 54	0.003349

The Montreal Neurological Institute (MNI) coordinates for the center position of the seed are placed in the bracket, right after the seeds’s name, whereas the MNI coordinates of the peak voxel of each target region are in a separate column. The relationships were significant (mass p-value FDR_C_, p < 0.05) with Bonferroni correction for multiple comparisons (p < 0.05).

**Figure 3:**
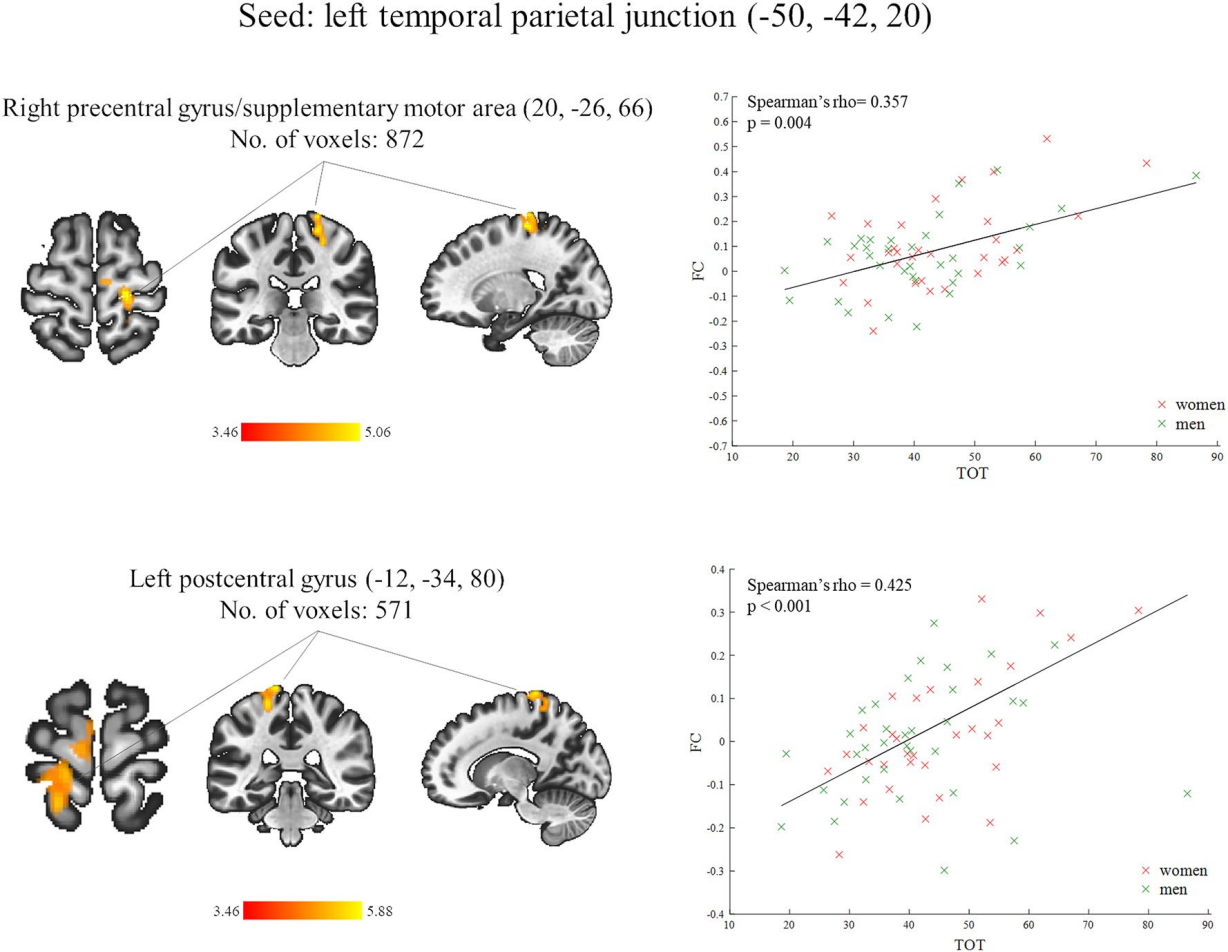
(Left) Brain regions demonstrating a significant positive association between the temporal order threshold (TOT) values and functional connectivity (FC) values after controlling for the influence of fluid intelligence (G_f_ factor), interaction between the TOT and G_f_ and gender. The left temporal parietal junction is the seed region with the Montreal Neurological Institute (MNI) coordinates of its center position placed in the bracket. The number of voxels is the size of the whole target region (in voxels 2 × 2 × 2 mm), and the name indicates the anatomical area the majority of the voxels belonged to. The color bars represent T scores. (Right) Scatter plots of functional connectivity (FC) parameters as a function of the temporal order threshold (TOT) at the peak voxel of each target region. The FC values for women are marked in red and for men in green. The black solid line represents a best fit for linear regression on a datasets with outliers (fit robust linear regression). The values of Spearman’s rho coefficients that measure the strength of association between the TOT and FC and their significance levels (p) are placed in the left upper corner of each scatter plot.

After controlling for the influence of G_f_ factor, the TOT × G_f_ interaction and gender, the TOT remained positively associated with the rsFC of the left TPJ with the right PreCG/SMA and the left PostCG (Fig. 3, Table 2, thresholded at the cluster-mass FDR-corrected level of p < 0.05 and Bonferroni corrected at p < 0.05). Neither the G_f_ nor the TOT × G_f_ interaction (after controlling for the remaining covariates) had significant influence on the functional connectivity of any of the seed regions corresponding to the left or right TPJ.

**Table 2:** Resting-state functional connectivity found correlated positively with the temporal order threshold (TOT) values after controlling for the influence of G_f_, TOT × G_f_ interaction and gender in all participants (n = 65).

	Seed region/MNI coordinates (x, y, z)	Main region in the target area	No of voxels	MNI coordinate (x, y, z)	p-value FDR_C_ p < 0.05 corr and Bonferroni p < 0.05 corr
1	Left temporal parietal junction (− 50, − 42, 20)	Right precentral gyrus/supplementary motor area	872	20, − 26, 66	0.000744
		Left postcentral gyrus	571	− 12, − 34, 80	0.001030

The Montreal Neurological Institute (MNI) coordinates for the center position of the seed are placed in the bracket, right after the seed’s name, whereas the MNI coordinates of the peak voxel of each target region are in a separate column. The relationships were significant (mass p-value FDR_C_, p < 0.05) with Bonferroni correction for multiple comparisons (p < 0.05).

The functional connections associated with the TOT values at FDR_C_ (p < 0.05) which failed to exceed the p < 0.05 Bonferroni threshold, are shown in the Supplementary Table S1. The Supplementary Fig. S1 presents the average SBC maps for each seed region, Fig. S2 contains the unthresholded statistical maps of the TOT-SBC relationship for each seed after controlling for the influence of gender whereas Fig. S3 shows the unthresholded statistical maps of the functional connectivity with the TOT when the influence of G_f_, TOT × G_f_ interaction and gender were controlled.

## Discussion

To the best of authors’ knowledge the present study is the first where rsFC was investigated in relation to the temporal order perception. We found the brain functional couplings of the left TPJ associated with visual TOT when the influence of gender was controlled: the higher the thresholded value, the stronger was rsFC of this region with the left PostCG, right PreCG, bilateral putamen and the right SMA (Fig. 2, Table 1). After controlling for the effect of G_f_, TOT × G_f_ interaction and gender, the TOT was still significantly related to the rsFC of the left TPJ with the right PreCG/SMA and the left PostCG (Fig. 3, Table 2). Our outcomes partially support the intrinsic models holding that a temporal resolution is a relatively stable feature of local neural circuits^47,48^. The regional changes in spontaneous functional connections, found here, may reflect the brain’s readiness and/or capacity to respond to rapid successive events.

In the current study the mean and median TOT values for visual stimuli, for all participants, were ca. 40 ms. (Fig. 1) which is perfectly in line with previous studies^4,5,18,49^. Following Davis et al.^27^ we focused on functional couplings of the 9 regions forming the left and right TPJs (see: Table 4). We found significant TOT—rsFC relationship for only 1 of 5 left-hemispheric TPJs, whose center position corresponds to the left superior temporal gyrus. Therefore, our outcomes confirm previous evidence demonstrating the left TPJ’s involvement in TOJs^27,30,32,50,51^.

So far, the left TPJ has been associated with correct TOJs only for the acoustic stimulation^30–32^. For example, the training-induced improvement in the auditory TOJ task coexisted with an increased left TPJ activity^30^. Our outcomes are consistent with these findings since we have demonstrated a positive relationship between the visual TOJ task correctness, expressed by the thresholded values, and the rsFC of the left (not right) TPJ (more specifically: the superior temporal gyrus). Therefore, we suggest that the TOJ accuracy may be reflected in the resting-state functional couplings between the left TPJ and the regions involved in voluntary movement control^52,53^ (the sensorimotor cortex, putamen and the SMA). In other words, these left TPJ’s interactions may be a relatively stable feature of baseline brain activity necessary to produce fast and accurate responses in the TOJ task.

The involvement of the motor and sensory brain areas is not surprising since our TOJ task has required motor control (similarly to the finger tapping). In the current study lower threshold values associated with reduced spontaneous synchronization between the left TPJ and the regions participating in movement execution and control may just reflect an increased readiness to learn fast and accurate motor reactions. This greater susceptibility of the somatosensory network to the experience-driven plastic changes may be considered as its relatively constant feature being reflected in basic, spontaneous brain activity. The proposed explanation of these outcomes is congruent with previous evidence on altered resting-state functional connections in the somatosensory cortex due to motor or perceptual training^54–57^. Specifically, in children or young adults decreased baseline functional connectivity of the sensorimotor network with the inferior parietal cortex or with the cortico-striato-cerebellar network, was associated with more efficient perceptual/procedural learning^54,55^.

When the TOT value is relatively low, i.e. two stimuli are separated by less than 20 ms., their order could be perceived, however, the strategies to integrate two rapid incoming stimuli into one percept are more likely used. In the visual modality two stimuli that appear rapidly at different positions may produce the impression of apparent motion^22,23^. In this case, temporal order can be obtained from the motion percept without an involvement of a central timing mechanism. Conversely, when TOT value is within normal range (> 20 ms.) the thresholded value may more closely reflect “pure” timing. The somatosensory cortex has been found to be involved in the timing tasks including the somatosensory temporal discrimination^58,59^ or finger tapping^60,61^ and also animal studies indicate the existence of temporally precise coding mechanism in the primary somatosensory cortex^62,63^. Like the somatosensory cortex, the putamen, belonging to the basal ganglia, as well as the SMA, have been involved in millisecond timing^60,64,65^. In view of this evidence the increased rsFC of the left TPJ with these regions, accompanied by higher TOT values, may be considered as a baseline brain activity preparing for TOJs more relied on a timing mechanism. It is also possible that the positive correlation of the TOT with the strength of the left TPJ’s rsFC, found in the current study, reflects the adaptation for the temporal properties of stimuli since this process has been found to be dependent on the primary sensory cortical areas^66,67^. On the other hand, “sensing” the motion while judging the temporal order probably activates the sensorimotor system more than not using this non-temporal strategy. Hence, during the TOJ task performance, an increased rsFC of the sensorimotor network could be expected and, consequently, this network would be more suppressed at rest.

In the current study the TOT was negatively correlated with the G_f_ factor which is consistent with the literature^14,16,18,68^. We did not observe any significant influence of the TOT–G_f_ interaction on the SBC maps of all our seed regions, including the left TPJ (left superior temporal gyrus) whose rsFC was significantly associated with the TOT when neither the G_f,_ nor the TOT–G_f_ interaction were controlled (Fig. 2, Table 1). Thus, the TOT—left TPJ’s rsFC relationship acted similarly in people with lower and higher G_f_ factor values. However, the G_f_ factor had some impact on the TOT—left TPJ’s spontaneous functional couplings. Specifically, when it was included in the regression model together with the TOT–G_f_ interaction, the TOT remained significantly associated with the left TPJ’s rsFC with two (the right PreCG/SMA and the left PostCG) from five clusters determined in the functional connectivity analysis without any covariates of interest (Fig. 2, Table 1). These outcomes may indicate that the left TPJ’s functional connections with the sensorimotor cortex and the right SMA, are more “purely” associated with the temporal threshold values. However, we are far from the conclusion that this rsFC pattern is specific for temporal order perception. Firstly, because we realize that a relatively high correlation between the TOT and G_f_ in our study reduces the sensitivity with which the unique contributions of any of these factors to the left TPJ’s rsFC can be evaluated. Secondly, not only fluid intelligence but also other cognitive factors such as attention^6,13,18^ or working memory^3,18^, which were not considered in the current study, may significantly affect the TOJ accuracy and thereby neural correlates of the TOJs including the resting-state functional connections associated with the TOT. Thirdly, we used only the TOJ task in one sensory modality which does not allow us to make any more general conclusions about the relationship between the TOT and spontaneous functional connectivity, especially, when we accept that the temporal threshold values are dependent on the physical features of the stimuli applied^7^.

By taking into account the effect of G_f_ factor and G_f_ × TOT interaction, we deprived the TOT of its significant relationship with rsFC between the left TPJ and bilateral putamen (Fig. 2, Table 1). The putamen is thought to respond to the stimuli that predict learned movements^69,70^ and it is activated when movements to non-rewarding stimuli must be suppressed^71^. Furthermore, the local morphology of putamen has been associated with fluid intelligence^72^. In the current study, TOJs, especially when they were made more automatically, can be considered as a sequence of learned movements (pressing response keys) evoked by stimuli (rapid light flashes). Since our TOJ task was two-alternative forced choice adaptive procedure, the participants had to, at least sometimes, inhibit their motor reactions. As we have mentioned earlier, temporal order tasks may be performed using a holistic strategy (perceptual integration of two visual stimuli to induce an impression of apparent motion) which fast become an automatic and effortless process. Considering that the ability to automatize a particular activity is thought to be a major aspect of intelligence^73,74^, we may assume that individuals with higher level of G_f_ factor are more likely to use this strategy in the TOJ task and the putamen may be involved in this process of learned motor reactions. Therefore, the relationship of the TOT with functional couplings between the left TPJ and this region could be affected by the G_f_ factor. It is, however, of note that in the present study the G_f_ (after controlling for the influence of TOT, TOT × G_f_ interaction and gender) was not significantly related to the TPJ’s rsFC with the right or left putamen.

The current study has some serious limitations. The generalization of our outcomes on the entire population is rather limited. Firstly, because of the relatively small sample size and, secondly, due to high scores obtained by the participants in the fluid intelligence tests designed for above-average intelligent people. Therefore, it is possible that current results are representative only for the individuals with higher cognitive abilities. We are also aware of the case that the rsFC variability in our study can be high and, thus, can significantly affect the results.

Considering further study directions, we intend to examine whether there is a similar relationship between rsFC patterns and the TOJs measured for other sensory modalities (auditory and tactile). Since in the current study the structures involved in the movements execution and control have been identified as being related to the TOT values (the sensorimotor cortex, putamen and the right SMA), it would be also advisable to compare the TOJ task results obtained with and without motor reactions. Actually, we plan to do so in the near future.

In conclusion, it appears to be reasonable to search for any connection between the resting-state brain activity and the temporal order threshold. We are not aware of any study that aims to examine this relationship. Our outcomes indicate that the regions thought to specifically involved in TOJs (the left TPJ) and the areas responsible for the movement execution and motor control (the sensorimotor cortex and the right SMA), may be prepared to cooperate in order to perform the visual TOJ task, even in the absence of any relevant stimulation. Spontaneous TOT-related interactions between the left TPJ and the somatosensory network may reflect the brain’s readiness to process the temporal aspects of incoming stimuli and to organize the motor responses to them in the subsecond time domain.

## Methods

### Participants

Out of 71 adolescents and young adults, recruited from regular high schools and high schools for gifted students in the area of Toruń, Poland, 65 (31 women, 34 men; mean age = 17.62 ± 1.07 year, age range: 16–19 years) comprised the study sample. Six persons were excluded from the study due to either excessive movement during the fMRI data acquisition or abnormally high (> 150 ms.) TOT value. There were no significant gender differences in the TOT value (z = − 1.215, p = 0.224).

All participants were right-handed and had normal or corrected-to-normal vision. They were in a good health, attended school regularly, reported no history or signs of neurological/psychiatric disorders and declared not taking any drugs affecting the central nervous system.

The study was approved by the local Bioethics Committee of the Nicolaus Copernicus University in Toruń, functioning at Collegium Medicum in Bydgoszcz, Poland, and was in line with the ethical principles of the 1964 Declaration of Helsinki. Each participant provided a written informed consent to take part in the study (in case of minors, an additional consent was obtained from their parents/caregivers). All participants received monetary remuneration for their time and effort.

### Procedures

All procedures were conducted in two days (within a week), during two, ca. 1.5-h sessions (with short breaks). The participants were randomly assigned to the following conditions: about 1/3 of them performed the entire TOJ task before the resting-state fMRI study, in another 1/3 the order of these procedures was reversed, and the remaining 1/3 had the first session of the TOJ task before and the other one after the fMRI study.

### TOT evaluation

The TOJ task was performed individually inside an acoustically-shielded chamber. Each stimulus consisted of a pair of 40-ms. light flashes emitted by the diodes mounted to the wall in front of the participants at two different locations (right and left) on a horizontal line. The diodes were placed 41 and 40 cm away from the fixation point (diode) on its left and right side, respectively. A participant seated behind the desk (75-cm high) on the chair (47-cm high), in a 161-cm distance from the fixation point and was asked to keep the head on the chin rest during the entire procedure. The viewing angle of the stimulation was 29.27°. The procedure was controlled using Raspberry Pi 3 Model B SBC (Bulk) equipped with a microcontroller Arduino Genuine Zero. Stimuli were generated by LED Maxim Integrated MAX16822BEVKIT+ controller and presented via white LED CREE diodes with a maximum luminance 350 lm. The two-alternative forced choice (2AFC) task was applied. Participants were asked to press the right or left key when the first diode in a pair flashed on the right or left side, respectively. Two response pads were connected to the computer with the optical fibers to ensure the least possible delay in signal transmission. A participant held one response pad in each hand and used the thumbs to report the answers. The TOJ task comprised 200 stimulus pairs and was divided into two sessions (100 trials per each). The TOT value was relatively stable across these two measurements. Since the TOTs were abnormally distributed across our participants in both sessions (Shapiro–Wilk’s test), the non-parametric Wilcoxon signed-rank test was used to compare the outcomes from these two measurements. The difference in the TOT between the first (median = 42.36 ms., TOT range: 15.88–115.83) and the second (median = 38.41 ms., TOT range: 18.2–70.75) session was not significant (z = − 1.80, p = 0.07). Therefore, the mean TOT value of the two measurements was included into the rsFC analysis. The inter-trial-interval was 3 s. The main task was preceded with 10 practice trials. As the result of the TOJ task, the TOT was determined individually in each participant. Similarly to previous studies, the TOT was defined as the ISI for which 75% correctness level was achieved^3,13,18^. TOT values were obtained based on the post hoc estimation of the psychometric curve from all the available trials collected during the TOJ task. The data were fitted by the Psignifit 4 toolbox implemented in Matlab^75^. The Supplementary Fig. S4 presents the psychometric curve fitted on the TOT values from all participants together whereas the Fig. S5 shows the psychometric curves, determined for each individual subject.

The scripts used in the study are available at: https://github.com/krzysztoftolpa/temporal-resolution. In the TOJ task the ISI was adjusted adaptively: it decreased after each correct response and increased after each incorrect one. We used an algorithm based on the Updated Maximum Likelihood (UML) implemented in the Matlab toolbox^76^.

### Fluid intelligence assessment

Fluid intelligence was evaluated with the use of two standardized tests: the Cattell Culture Fair Intelligence Test (CFT-3)^77^ in the Polish adaptation by Matczak and Martowska^78^ and the Raven’s Advanced Progressive Matrices (RAPM)^79^ in the Polish adaptation of Jaworowska^80^. Both the CFT-3 and RAPM require inductive reasoning based on a geometric material and is designed for above-average intelligent people. The CFT-3 consists of two analogous parts, A and B. Each part includes four tests: (1) Series, i.e. 13 three-element sequences, each of them should be completed with the fourth pattern, selected from 6 available options, (2) Classification, where the material includes 14 sets of 5 pictures from which 2 are different in terms of a single aspect and the task is to identify those mismatched pictures; (3) Matrices: 13 matrices are arranged according to two different criteria; it is required to select 1 of 6 available patterns—the one that best matches each matrix; and (4) Topological inference which is made based on 10 pictures showing the arrangements of geometrical figures; the task is to choose (from 5 options) the pattern that replicates a given arrangement. The number of correct responses in the whole test was calculated (max. 50 points in each part resulting in total of max. 100 points). The RAPM consists of the training part with 12 tasks and the proper test composed of 36 tasks. The tasks are in a form of incomplete patterns (matrices) which should be completed with one of 8 available elements. The number of correct responses in the test was calculated (max. 36 points). The principal component analysis (PCA) was used to extract the G_f_ factor based on the RAPM and the CFT-3 outcomes. The G_f_ factor explained 83.57% of total variance in the RAPM and the CFT-3 scores (eigenvalue = 1.67, see: Table 3 for details).

**Table 3:** Descriptive statistics and correlations (Pearson's r) for the intelligence tests used to extract the G_f_ factor (n = 65).

	RAPM	CFT-3	G_f_ factor
M	23.98	61.06	0
SD	5.95	8.08	1
Min/max	10/35	39/75	− 2.51/1.69
Skewness	− 0.41	− 0.45	− 0.27
Kurtosis	− 0.6	− 0.04	− 0.68
r (G_f_)	0.91	0.91	
r (CFT-3)	0.67		

RAPM, Raven’s Advanced Progressive Matrices; CFT-3, Cattell Culture Fair Intelligence Test.

### Resting-state fMRI data acquisition

Resting-state fMRI data were acquired at the Interdisciplinary Centre for Modern Technologies, Toruń, Poland, using a GE Discovery MR750 3 Tesla MRI scanner (General Electric Healthcare) equipped with a standard 8‐channel head coil. The parameters of EPI sequence were the following: time of repetition (TR) = 2000 ms.; time of echo (TE) = 28 ms.; flip angle (FA) = 900, voxel size = 3 × 3 × 3.3 mm; imaging matrix = 64 × 64; no of slices = 44; 300 data points, 8 dummy scans. Participants were instructed to relax during scanning with their eyes open and not to think of anything in particular. A structural T1 MR sequence had the following parameters: TR = 7832 ms.; TE = 3.02 ms.; inversion time (TI) = 400 ms.; flip angle (FA) = 120; field of view (FOV) = 24 × 24 cm; matrix = 256 × 256; voxel size = 0.9375 × 0.9375 × 1 mm; pixel bandwidth = 244.141 Hz/pix; no of slices = 252.

### Resting-state fMRI data pre-processing

The fMRI data were preprocessed according to the pipeline applied in CONN toolbox ver. 20b^81^. The steps included: slice time correction, motion correction, scrubbing, linear detrending, band-pass filtering (0.01 Hz < f < 0.1 Hz), co-registration to individual T1 anatomical scans, spatial normalization to MNI space, and spatial smoothing (6 mm Gaussian kernel). Each subject’s structural scan was segmented into gray matter, white matter, and cerebrospinal fluid (CSF) tissue classes using the unified segmentation approach implemented in SPM12 (https://www.fil.ion.ucl.ac.uk/spm/software/spm12/). In addition, the Artifact Detection Tool (https://www.nitrc.org/projects/artifact_detect) was used to measure motion artifacts in all participants. Linear regression of confounding effects was applied including: mean signal from white matter and CSF, motion parameters obtained in the realignment step, volumes that showed movement exceeding 0.5 mm from the scrubbing step.

Definition of seed regions of interest. Nine 5-mm radius spherical seed regions were produced using the Marsbar toolbox https://marsbar-toolbox.github.io/index.html. The center positions of these seeds were taken from the work by Davis et al^27^. We decided to include to our analysis all regions corresponding with the left or right TPJ activated by the visual TOJ tasks described in this paper. The list and coordinates of the seeds used in our analysis are presented in Table 4.

**Table 4:** The list of the sources included in the resting-state functional connectivity analysis.

Seed/source	MNI coordinates (x, y, z)
Left temporal parietal junction	− 66, − 40, 10
Left temporal parietal junction	− 66, − 38, 24
Left temporal parietal junction	− 50, − 48, 10
Left temporal parietal junction	− 50, − 42, 20
Left temporal parietal junction	− 34, − 48, 12
Right temporal parietal junction	58,− 40, 24
Right temporal parietal junction	60,− 52, 16
Right temporal parietal junction	64,− 50, 14
Right temporal parietal junction	66,− 44, 20

The Montreal Neurological Institute (MNI) coordinates of the central positions of the seeds were taken from the work by Davis et al.^27^, who found nine regions belonging to either the left or right temporal parietal junction, activated by the fMRI visual temporal order judgment tasks.

### Statistical analysis

The Spearman's rank-order (Spearman’s rho) correlation between the TOT and G_f_ factor was calculated using SPSS ver. 27 software. The first- and second-level analysis of resting-state fMRI outcomes were conducted using the CONN toolbox (ver. 21a). The first-level whole-brain Seed–Based Connectivity (SBC) maps were obtained by estimating Fisher-transformed correlation coefficients for each region of interest and for each participant. For all participants (n = 65), to calculate bivariate correlations between the connectivity strength and the TOT values, the seed-to-voxel correlation coefficients (Pearson’s r correlation coefficients) were computed and converted to normally distributed z-scores using Fisher’s transform. A second-level random effects analysis was applied to produce within-group statistical parametric maps (SPMs) with one T value for each voxel in the map characterizing the effect of interest (correlation between the TOTs and seed-based rsFC) at each location.

Previous studies have revealed gender-related differences in rsFC^82–86^. For example, Biswal et al.^86^ found that women, relative to men, exhibited greater rsFC in the posterior cingulate gyrus, medial prefrontal cortex and inferior parietal lobe but weaker connectivity in the dorsal anterior cingulate cortex, insula, superior temporal gyrus and occipital regions. Therefore, gender was included in the second-level SBC model as a covariant of no interest. As a result, the following SBC model was employed: y = b0 + (b1 * TOT) + (b2 * gender).

Since in the present study the G_f_ factor was significantly associated with the TOT, an additional second-level SBC model (y = b0 + (b1 * TOT) + (b2 * G_f_ factor) + (b3 * (TOT * G_f_ factor)) + (b4 * gender)) was run to explore the TOT × G_f_ interaction (while controlling for the influence of TOT, G_f_ and gender) as well as the TOT effect after controlling for the influence of G_f_, interaction between the TOT and G_f_ and gender. This SBC analysis was performed separately for each predefined seed region.

Cluster-level inferences based on randomization/permutation analyses were made^87^. Specifically, the cluster sizes were numerically estimated using multiple (n = 1000) randomization/permutation iterations of the original data. For each of these iterations, the statistical parametric map of T values was computed and thresholded in the same way as in the original data. SPMs were thresholded at the voxel level at p < 0.001, uncorrected, and, then, the cluster-mass FDR-corrected level of p < 0.05 using the standard Benjamini and Hochberg’s FDR algorithm (clusters with larger mass than what we could reasonably expect under the null hypothesis). At the end, the Bonferroni method at p < 0.05 to account for the effect of comparisons between multiple regions of interest was applied.

## Supplementary Information

Supplementary Information.

## Data Availability

The datasets used and/or analysed during the current study available from the corresponding author on reasonable request.
