# Supplementary material for: Resting-state fMRI functional connectivity of the left temporal parietal junction is associated with visual temporal order threshold
Source: Sci Rep. 2022 Sep 24;12:15933. doi: 10.1038/s41598-022-20309-1 (PMC9509386; doi:10.1038/s41598-022-20309-1)

Table **S1**. Resting-state functional connectivity found correlated positively with the temporal order threshold (TOT) values after controlling for the influence of G_f factor, the TOT × G_f factor interaction and **gender** in all participants (n = 65). The Montreal Neurological Institute (MNI) coordinates for the center position of each seed are placed in the brackets, right after the seeds' names, whereas the MNI coordinates of the peak voxel of each target region are in a separate column. The significant relationships (mass p-value FDR_C, $p < 0.05$) which failed to survive Bonferroni correction for multiple comparisons ($p < 0.05$) are shown. If the brain side of the target region is not stated, it was found in both hemispheres.

	Seed region/MNI coordinates (x, y, z)	Main region in the target area	No. of voxels	MNI coordinates (x, y, z)	p-value FDR_C $p < 0.05$ corr.
1.	Left temporal parietal junction (-50, -42, 20)	Left putamen	2**78**	-32, -6, -6	0.009**073**
		Right putamen	26**6**	26, -2, -2	0.009**073**
		Precuneus/left precentral gyrus	1**90**	-16, -38, 48	0.0**13406**
		Precuneus	1**63**	1**6**, -40, 4**6**	0.0**20465**

Figure S1. Average unthresholded seed-to-whole-brain connectivity maps calculated using non-parametric statistics. A reference BOLD signal time-course from each seed indicated at the top of each figure is correlated with the BOLD time-course for all gray-matter voxels in the image, revealing areas to which the seed is functionally connected (hot colors = strong positive correlation, cool colors = weak or negative correlation). The seed region names and the MNI coordinates (x, y, z) of each seed center position (in brackets) are placed at the top of each figure.

LEFT TEMPORAL PARIETAL JUNCTION (-66, -40, 10)

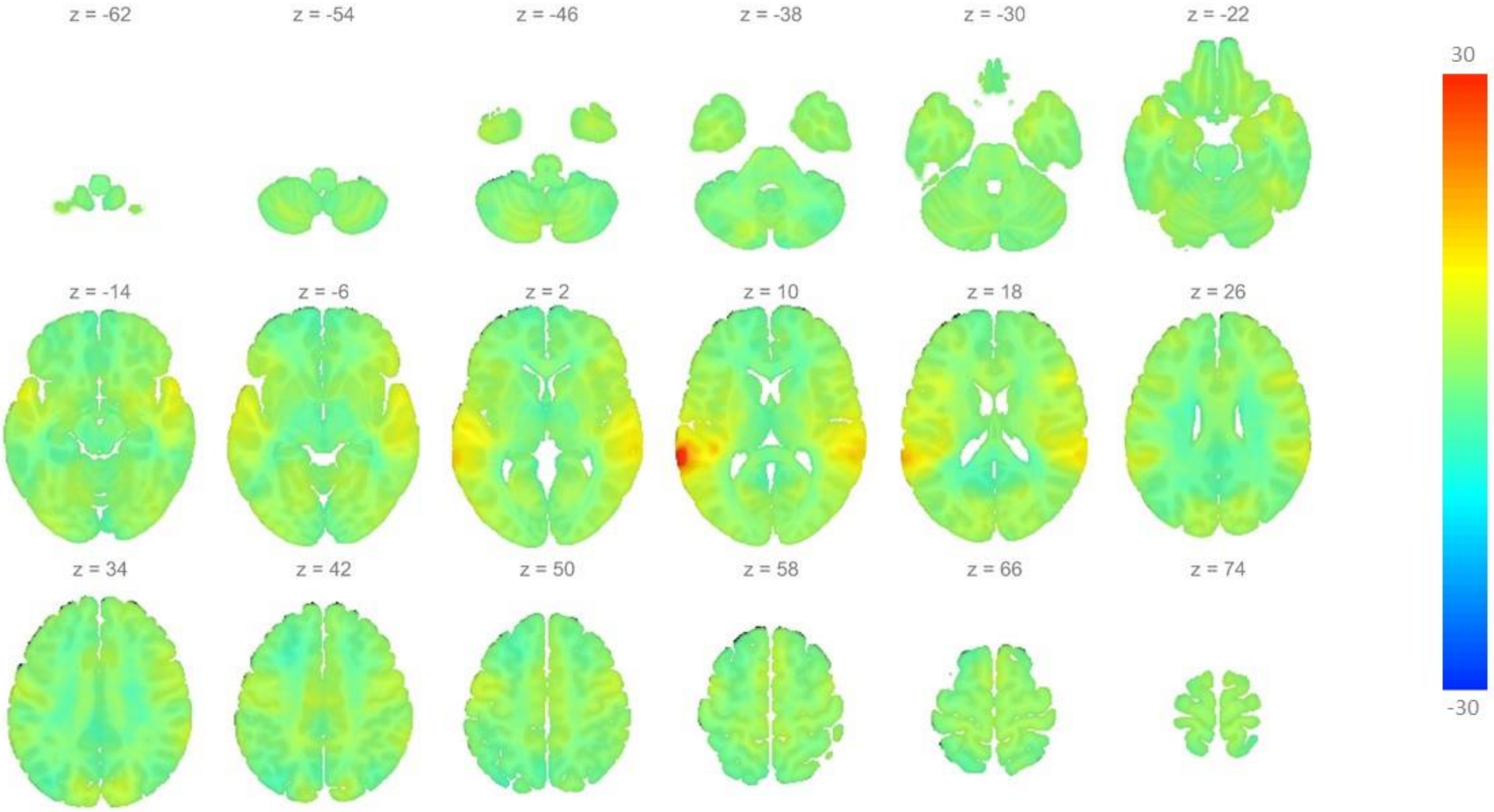

LEFT TEMPORAL PARIETAL JUNCTION (-66, -38, 24)

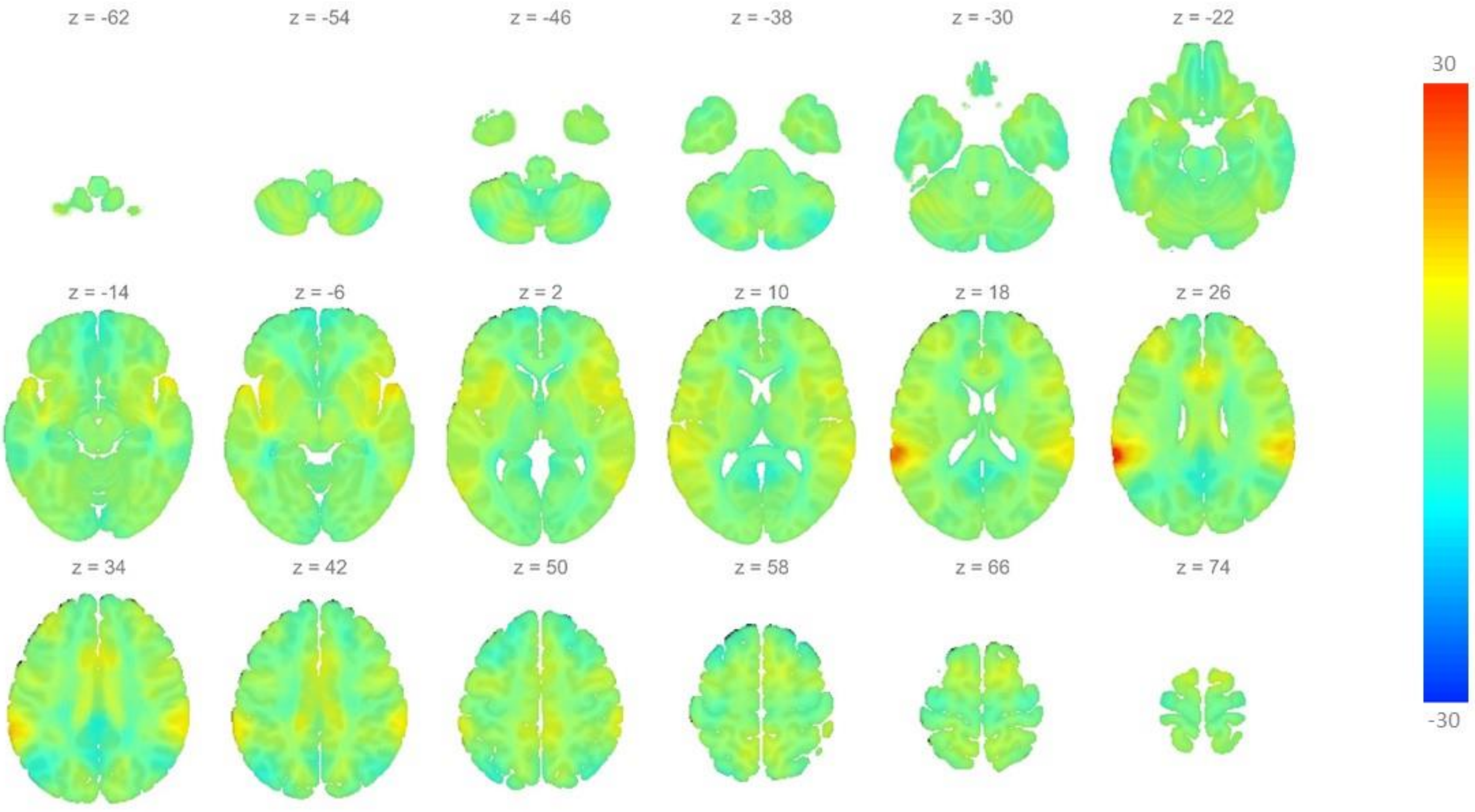

LEFT TEMPORAL PARIETAL JUNCTION (-50, -48, 10)

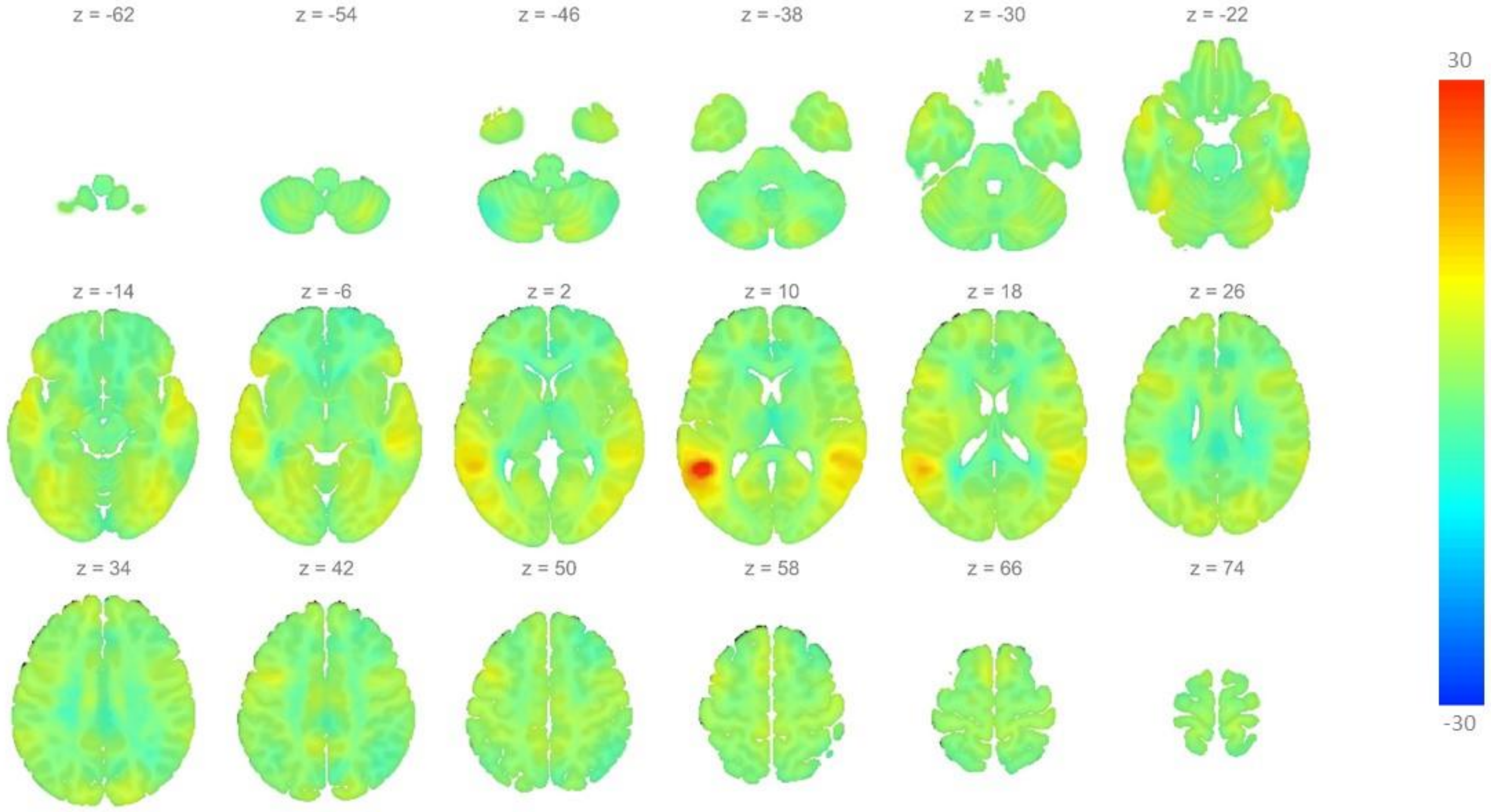

LEFT TEMPORAL PARIETAL JUNCTION (-50, -42, 20)

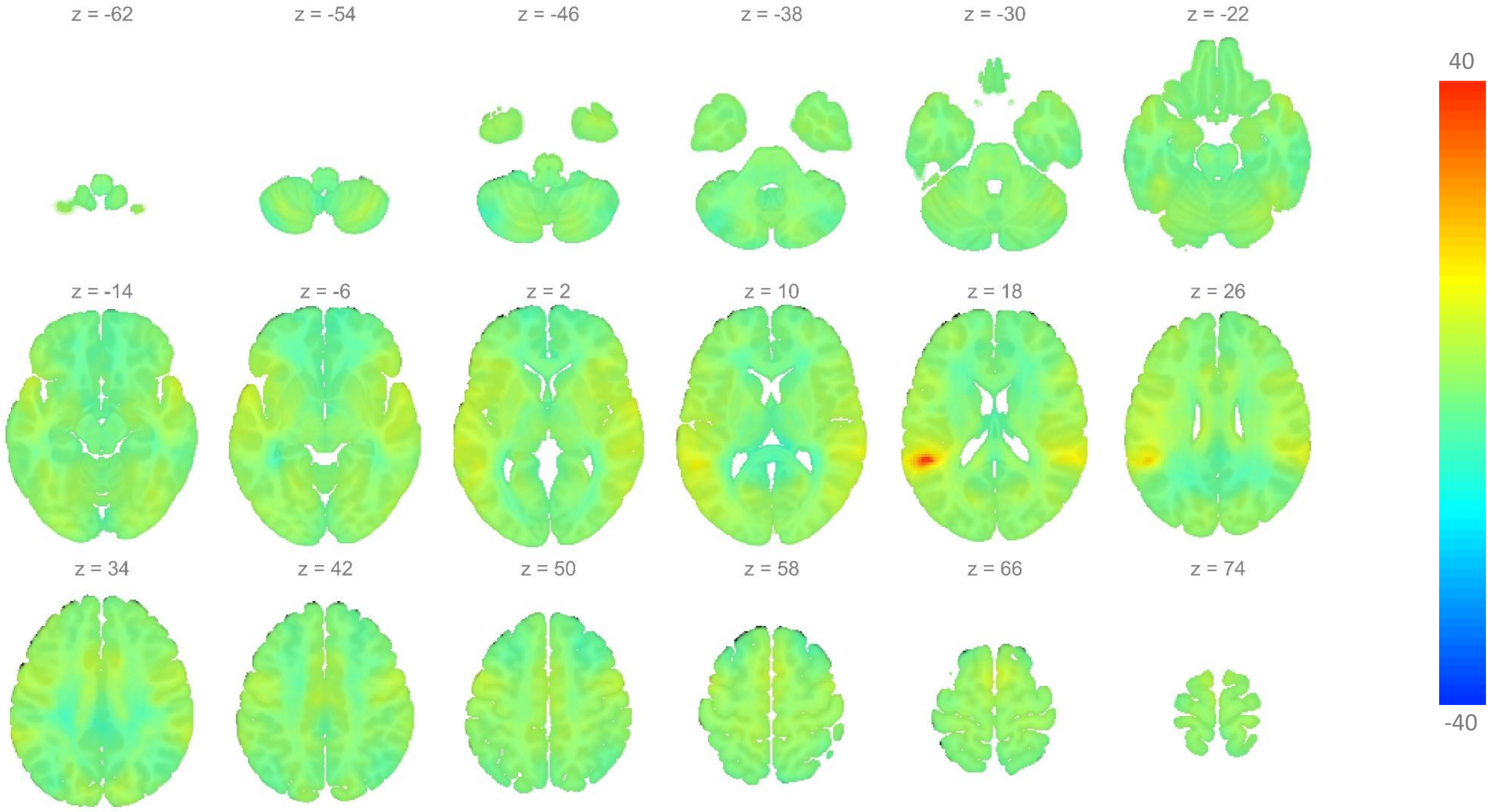

LEFT TEMPORAL PARIETAL JUNCTION (-34, -48, 12)

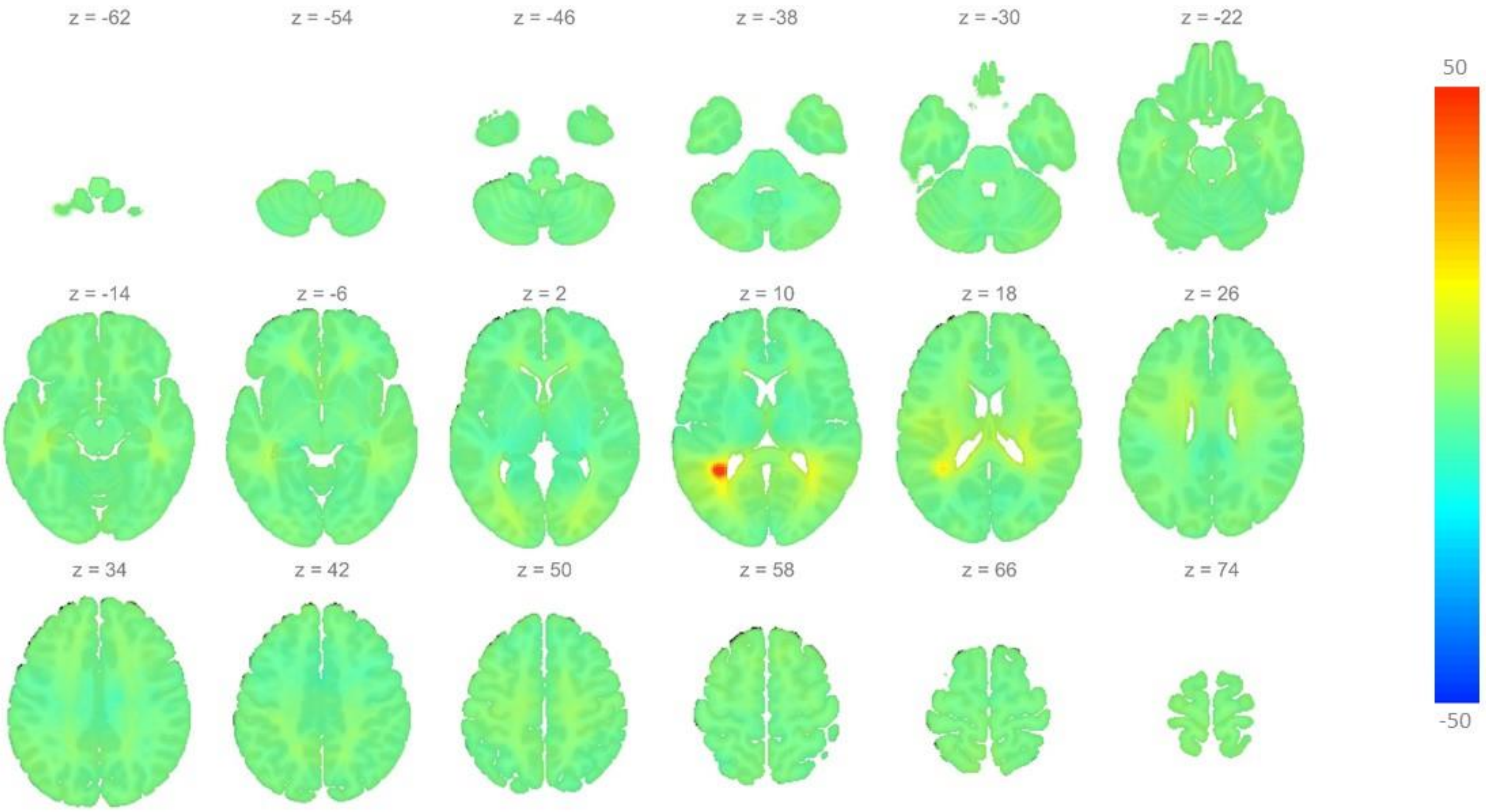

RIGHT TEMPORAL PARIETAL JUNCTION (58, -40, 24)

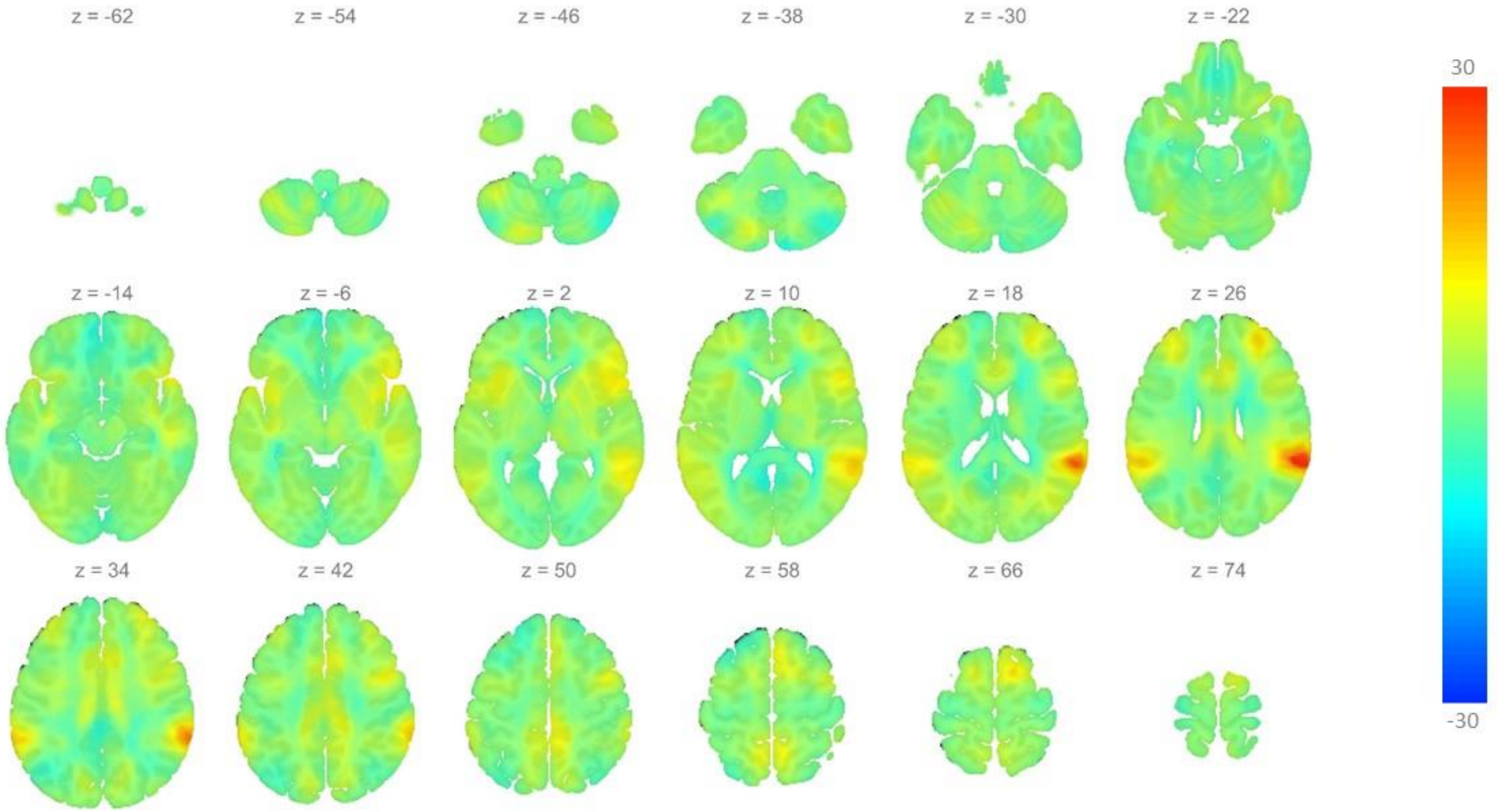

RIGHT TEMPORAL PARIETAL JUNCTION (60, -52, 16)

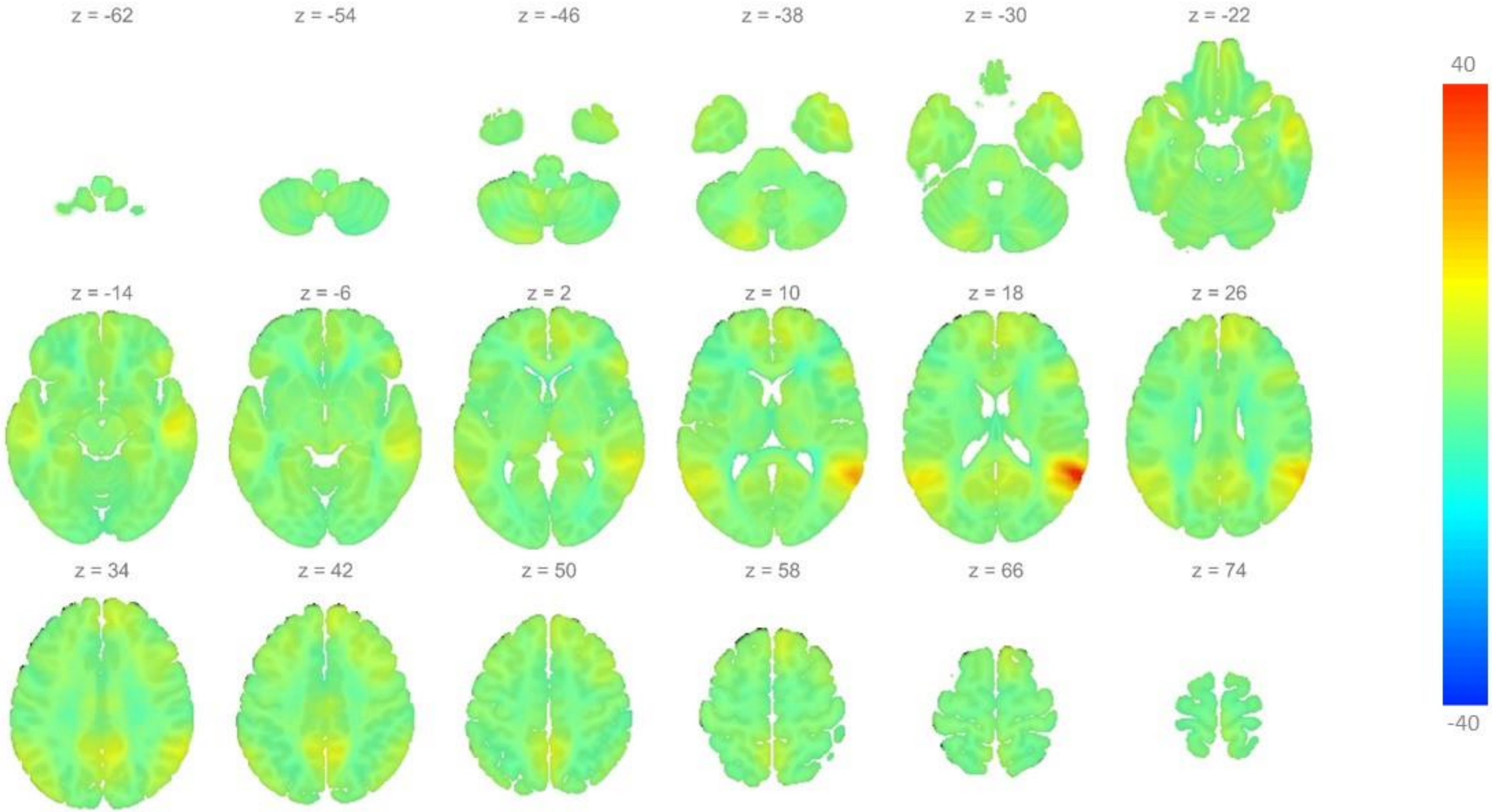

RIGHT TEMPORAL PARIETAL JUNCTION (64, -50, 14)

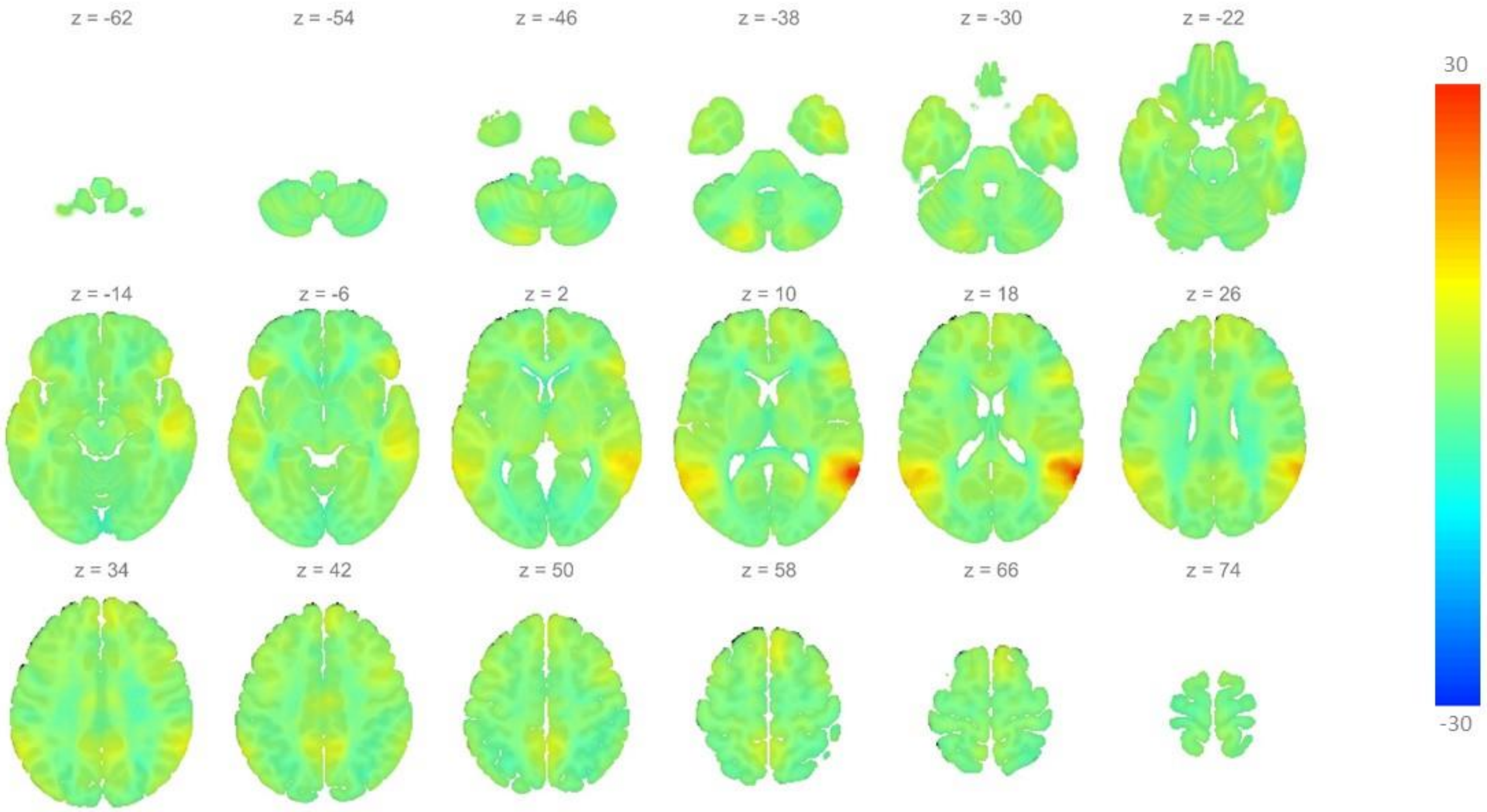

RIGHT TEMPORAL PARIETAL JUNCTION (66, -44, 20)

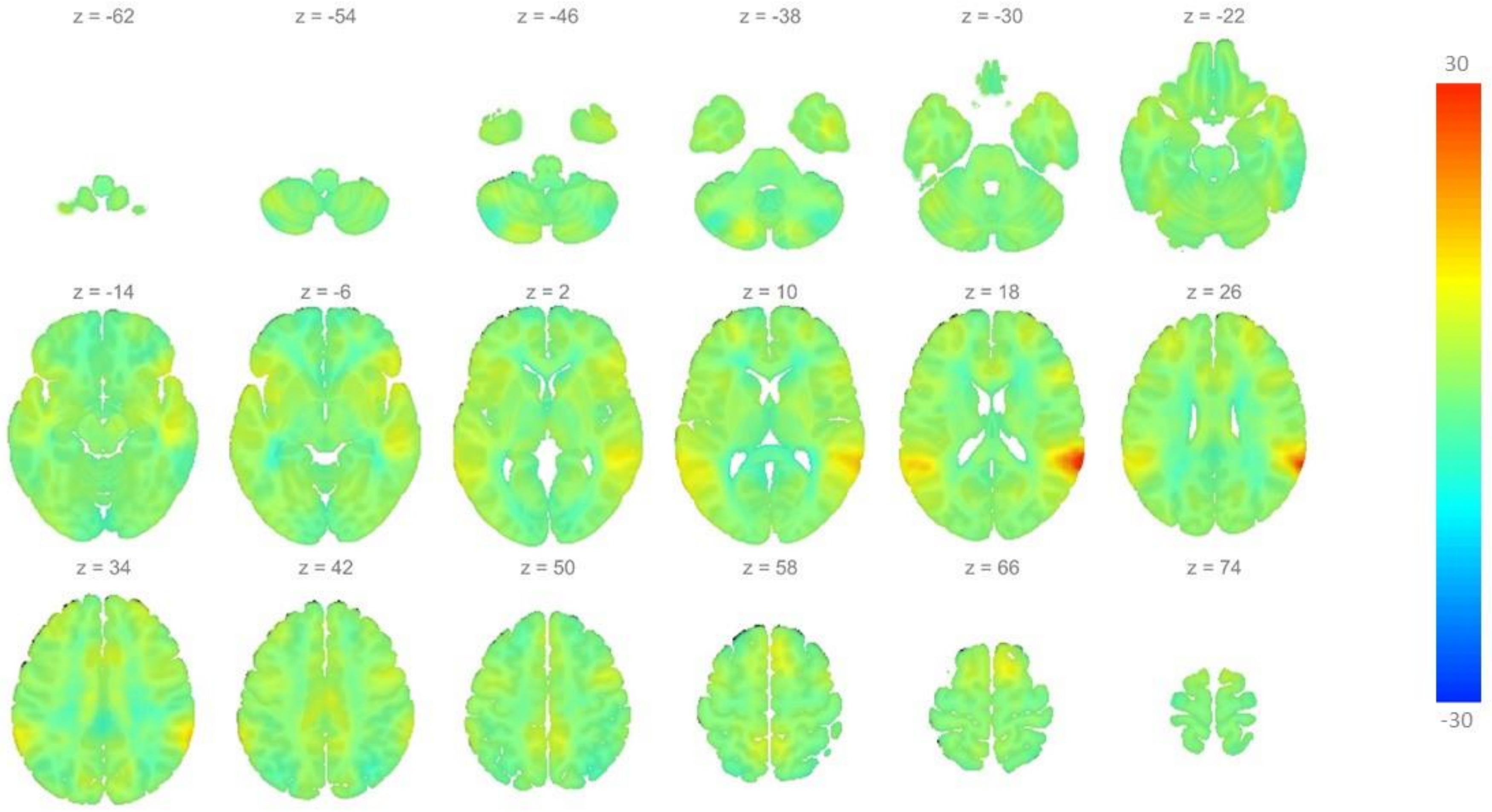

Figure S2. Untresholded statistical seed-based functional connectivity maps (created using permutation non-parametric statistics) related to the temporal order threshold (TOT) for each seed region included in the analysis. The map shows areas where there is a positive (hot colors) or negative (cold colors) correlation with TOT defined as a continuous variable for the whole group (n = 65) **while controlling for gender.** The seed region names and the MNI coordinates (x, y, z) of each seed center position (in brackets) are placed at the top of each figure.

LEFT TEMPORAL PARIETAL JUNCTION (-66, -40, 10)

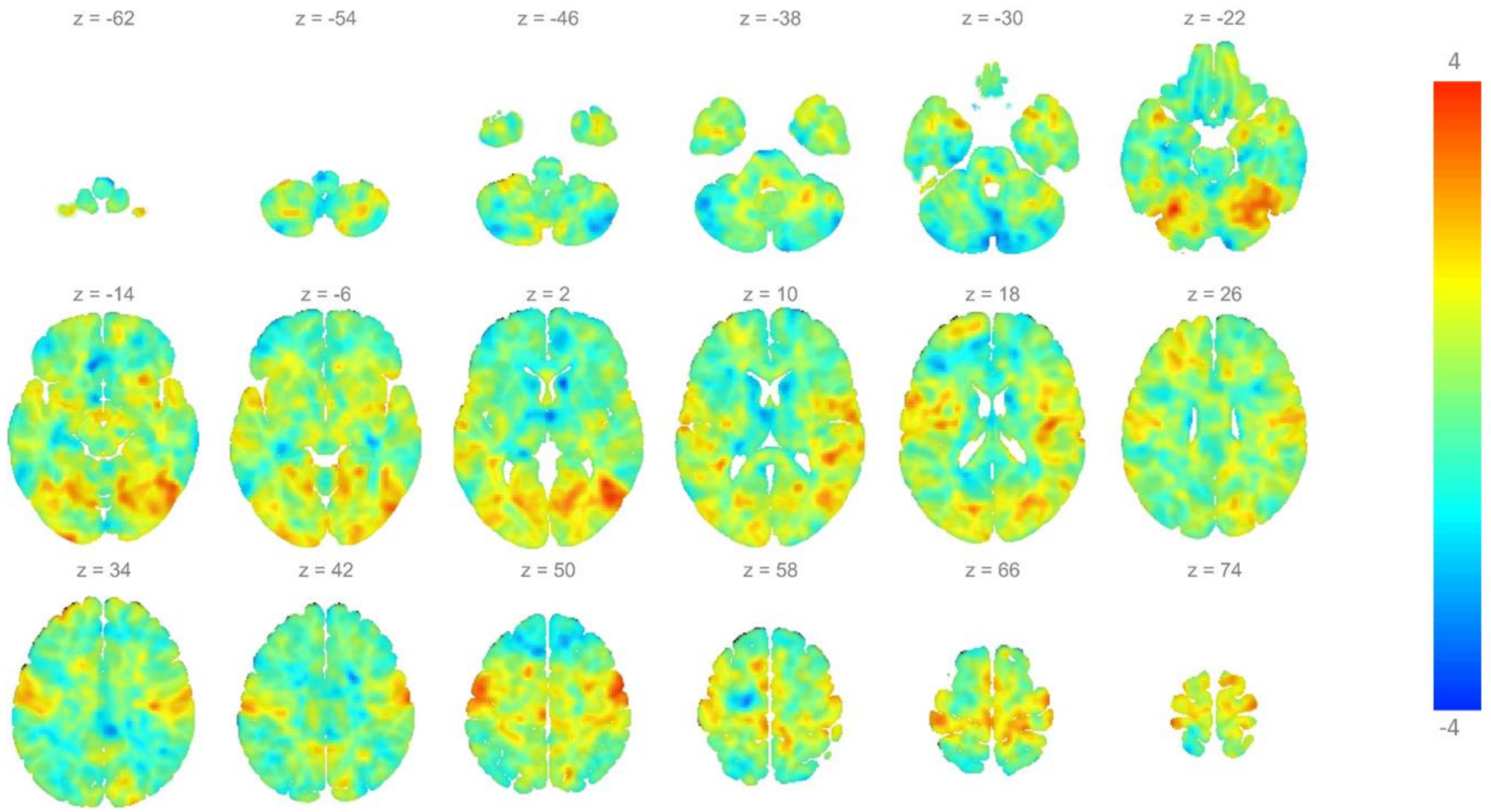

LEFT TEMPORAL PARIETAL JUNCTION (-66, -38, 24)

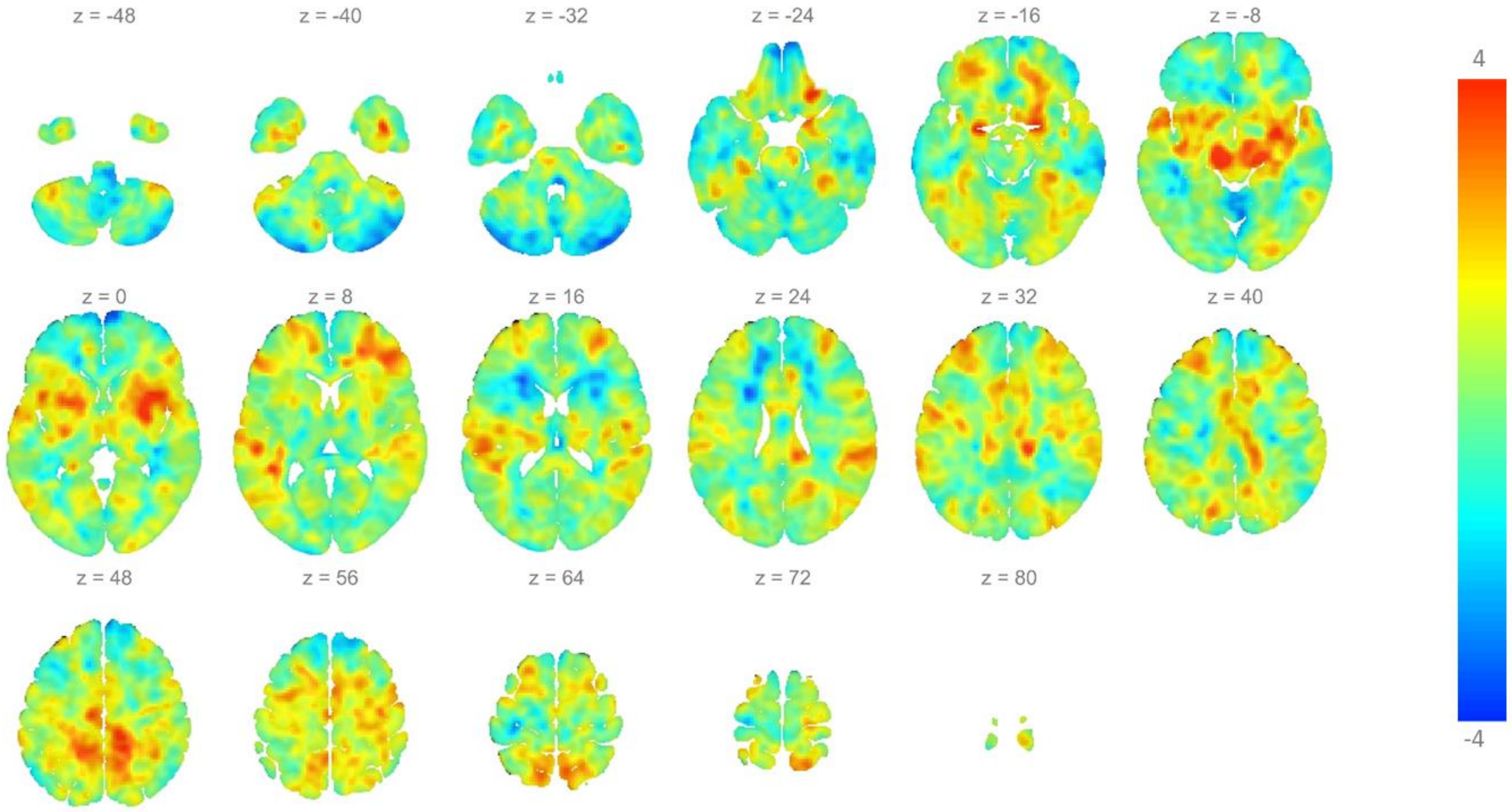

LEFT TEMPORAL PARIETAL JUNCTION (-50, -48, 10)

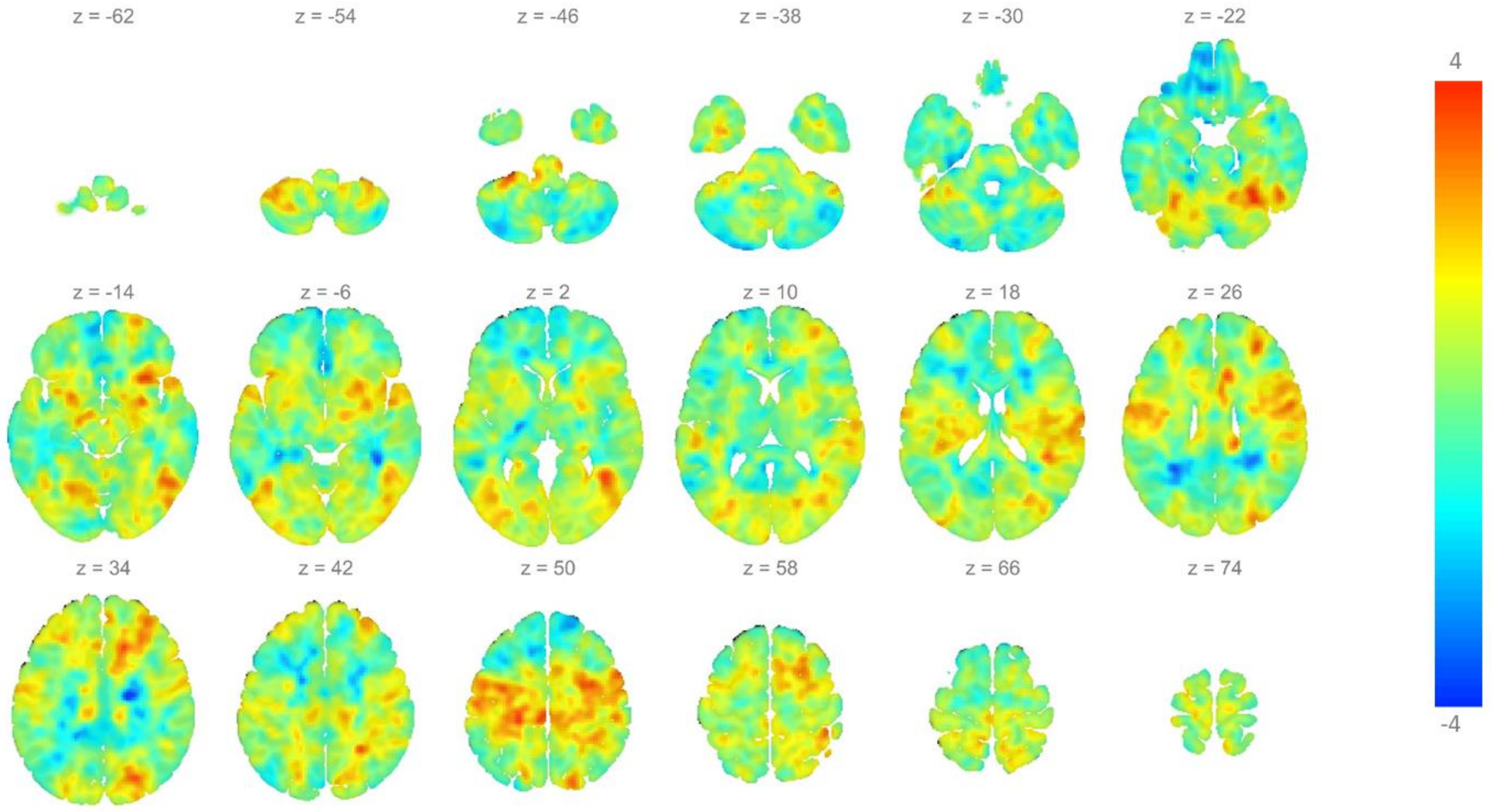

LEFT TEMPORAL PARIETAL JUNCTION (-50, -42, 20)

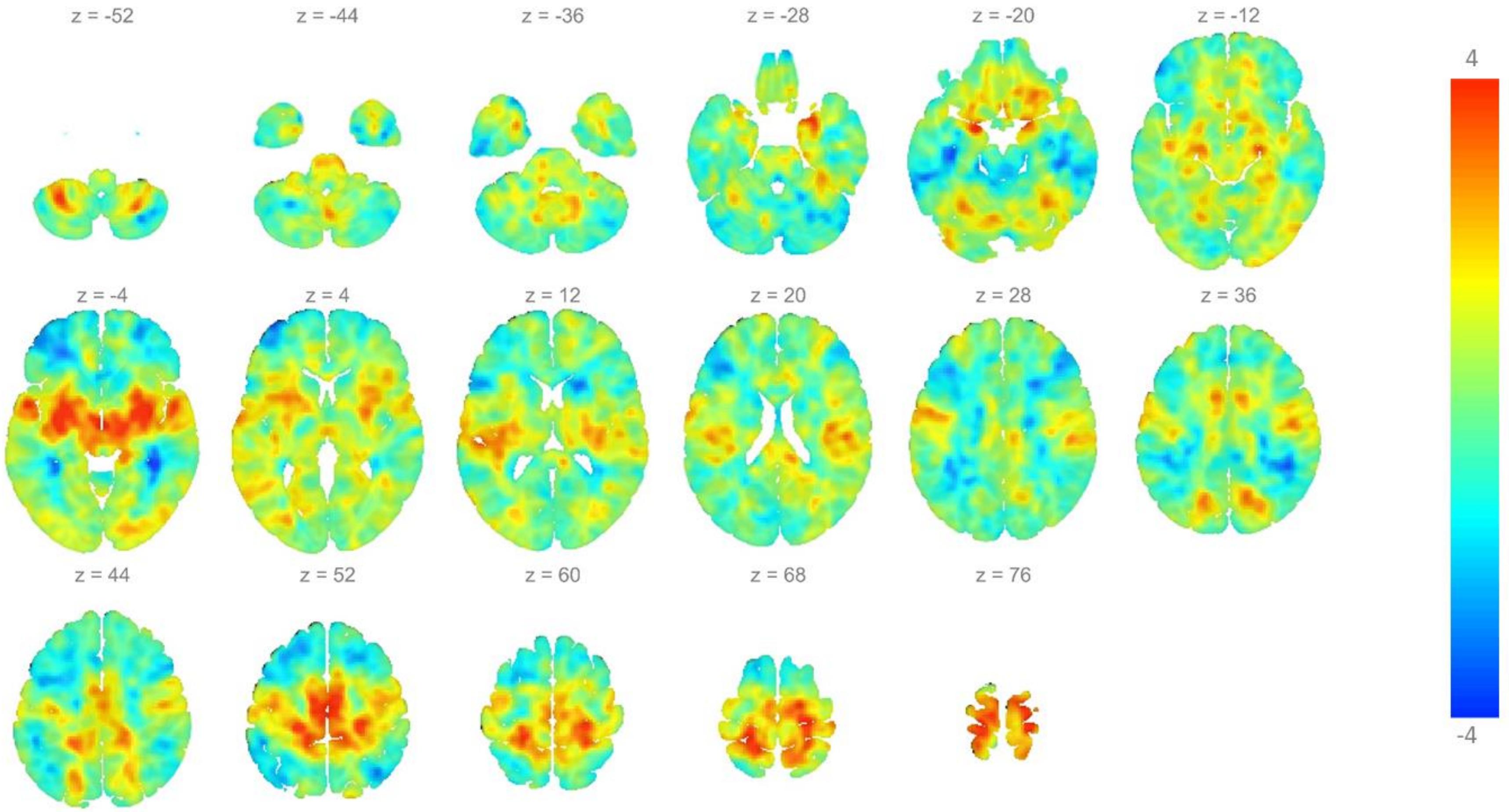

LEFT TEMPORAL PARIETAL JUNCTION (-34, -48, 12)

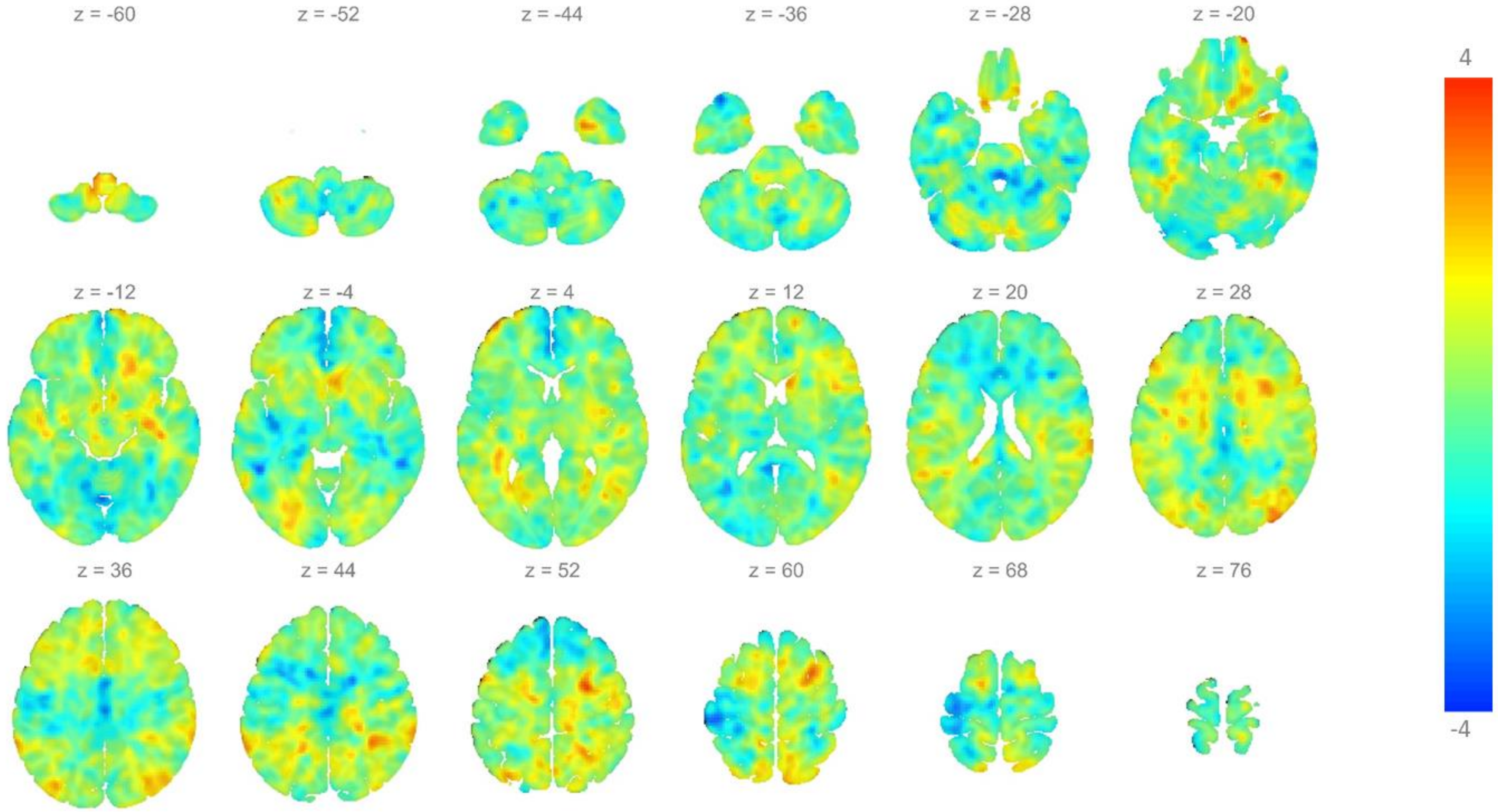

RIGHT TEMPORAL PARIETAL JUNCTION (58, -40, 24)

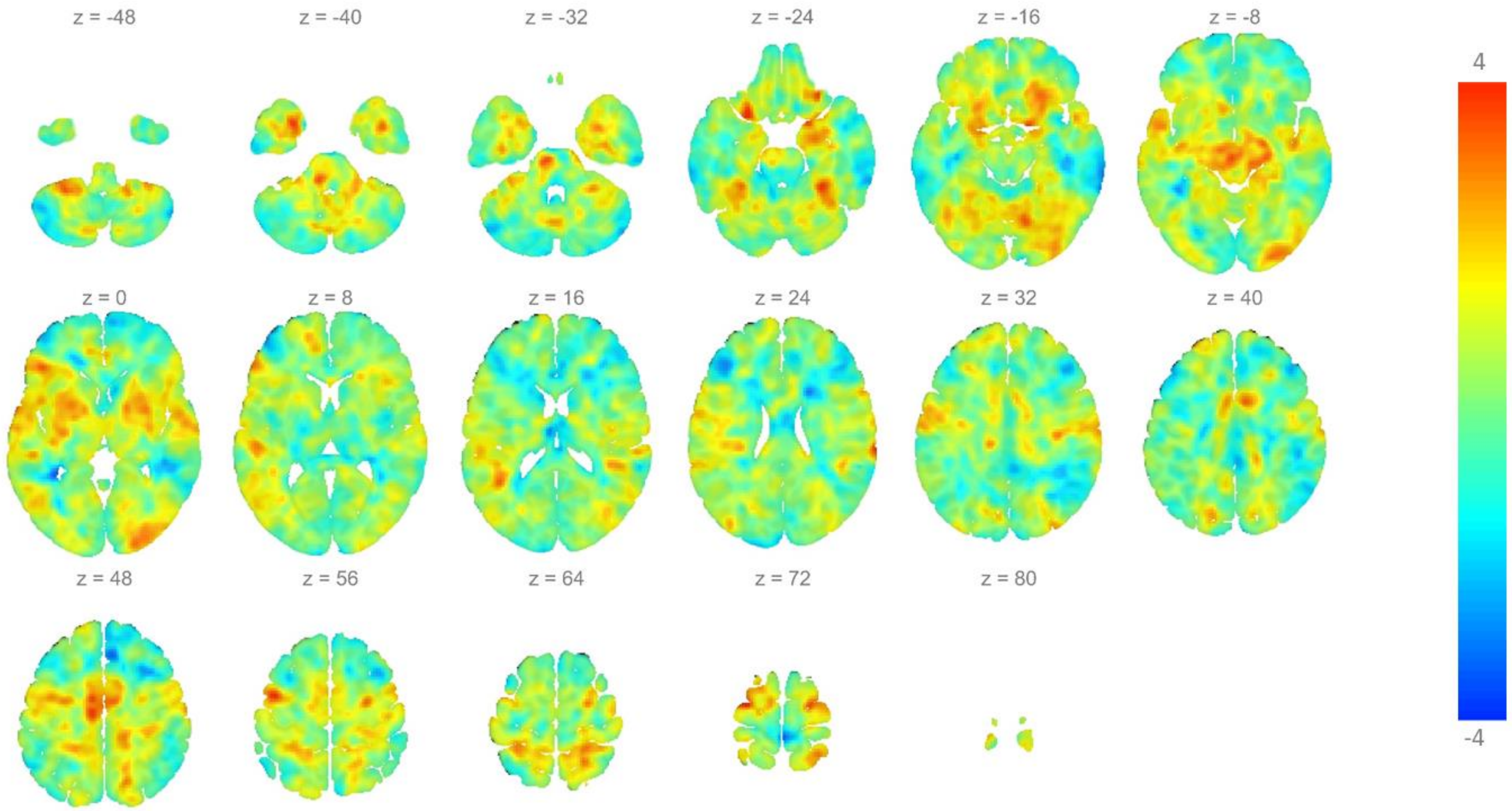

RIGHT TEMPORAL PARIETAL JUNCTION (60, -52, 16)

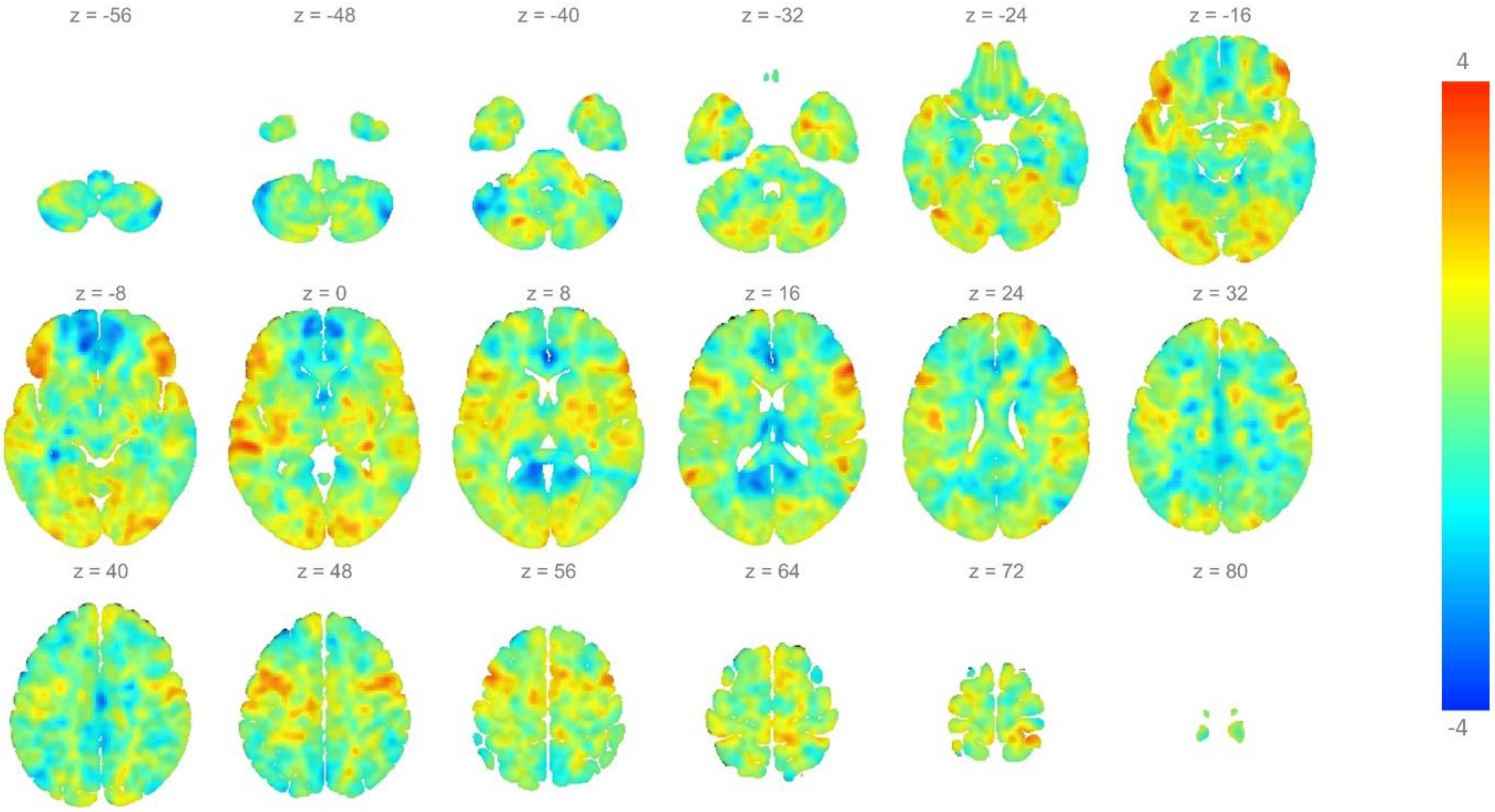

RIGHT TEMPORAL PARIETAL JUNCTION (64, -50, 14)

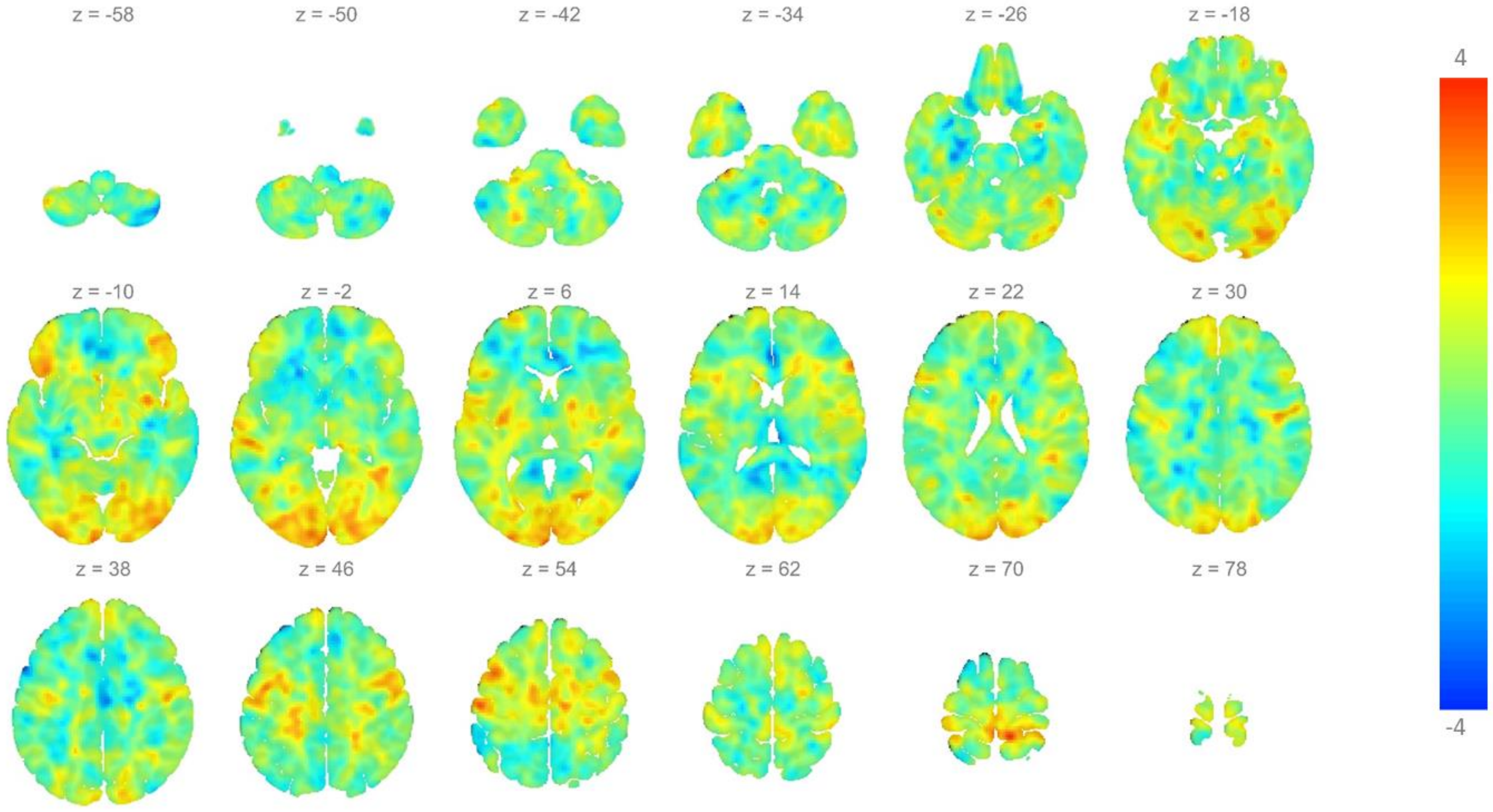

RIGHT TEMPORAL PARIETAL JUNCTION (66, -44, 20)

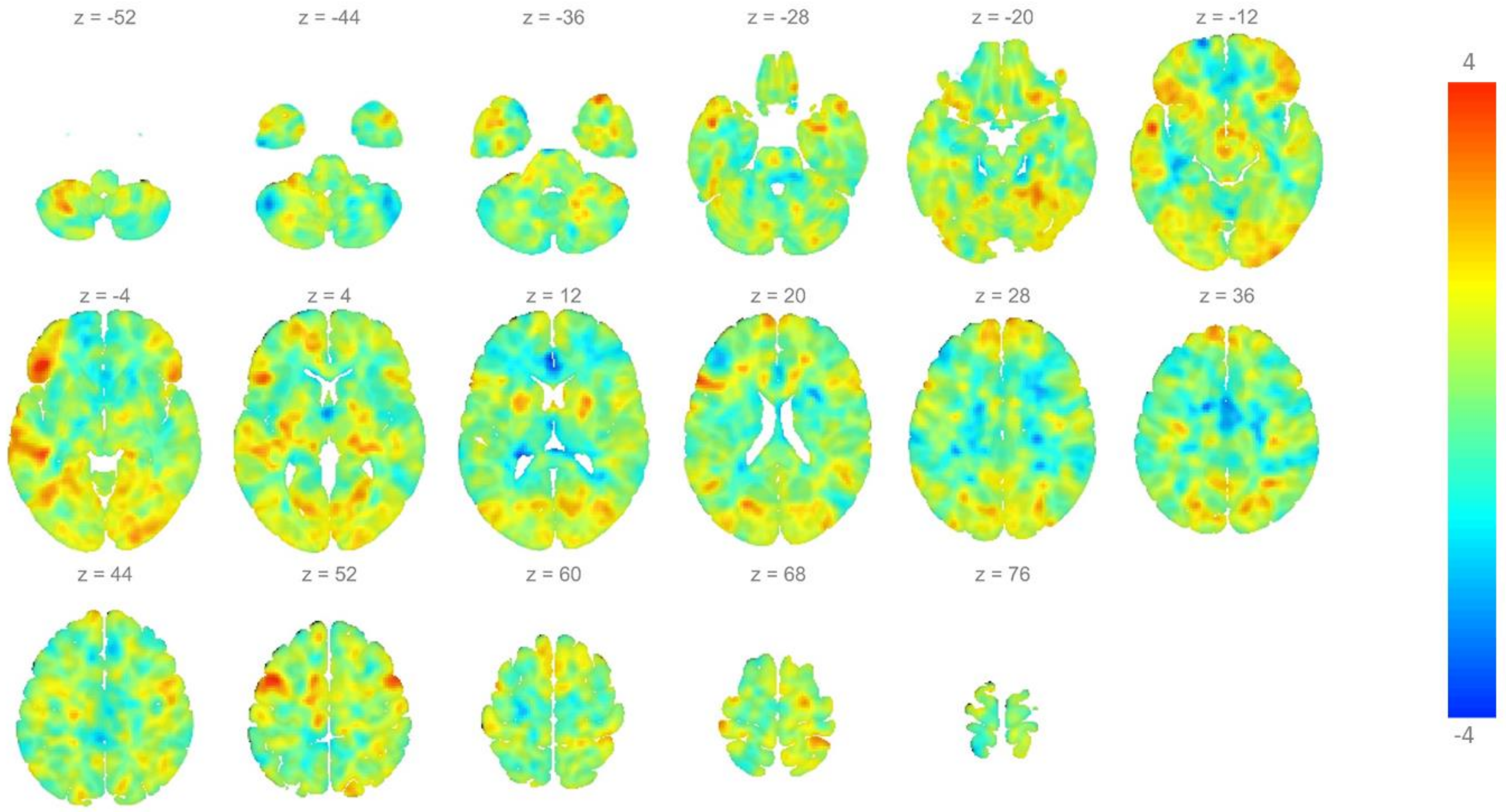

Figure S3. Untresholded statistical seed-based functional connectivity maps (created using permutation non-parametric statistics) related to the temporal order threshold (TOT) for each seed region included in the analysis. The map shows areas where there is a positive (hot colors) or negative (cold colors) correlation with TOT defined as a continuous variable for the whole group (n = 65) while controlling for the influence of fluid intelligence (G_f factor), TOT- G_f interaction **and gender**. The seed region names and the MNI coordinates (x, y, z) of each seed center position (in brackets) are placed at the top of each figure.

LEFT TEMPORAL PARIETAL JUNCTION (-66, -40, 10)

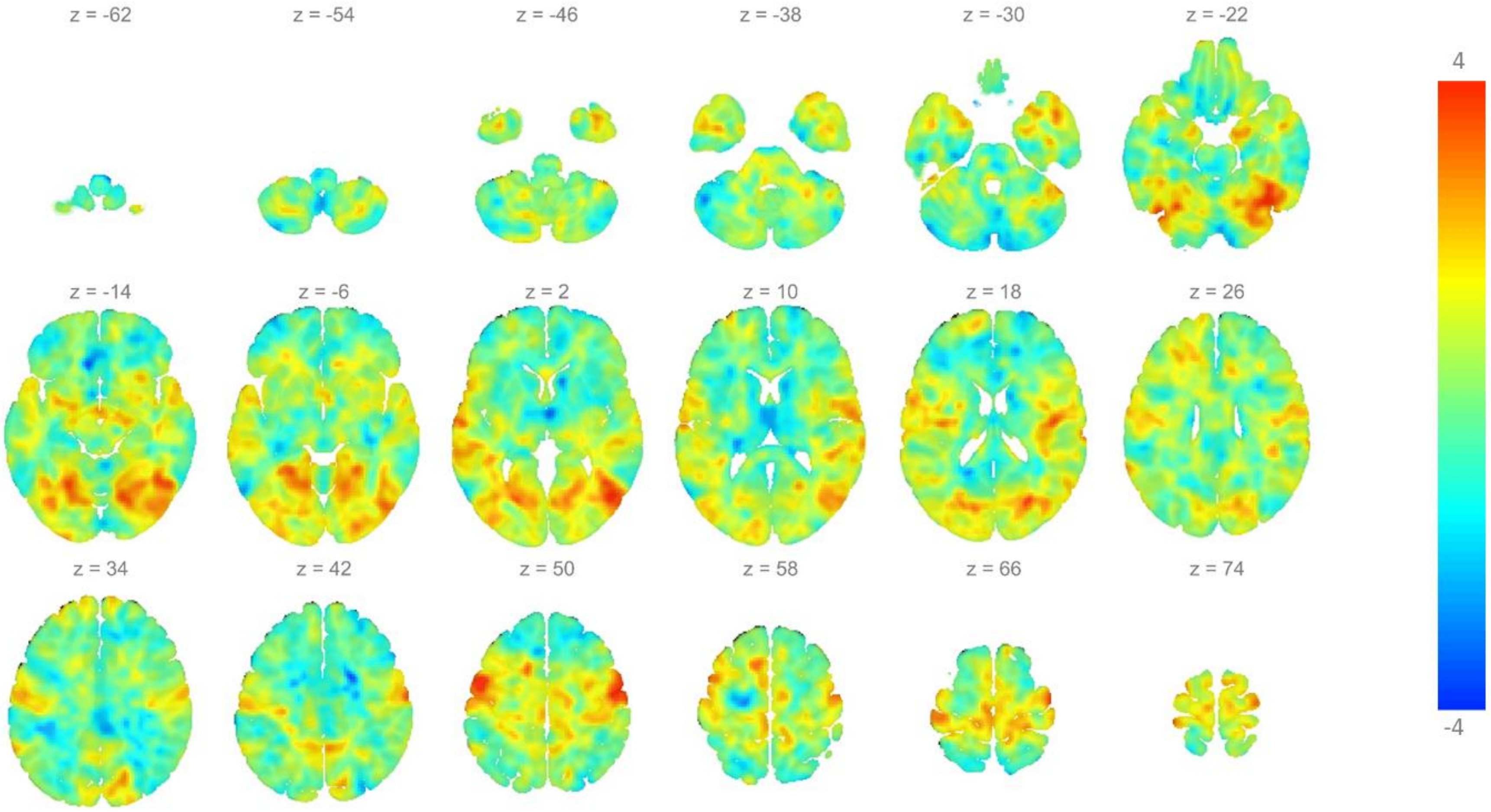

LEFT TEMPORAL PARIETAL JUNCTION (-66, -38, 24)

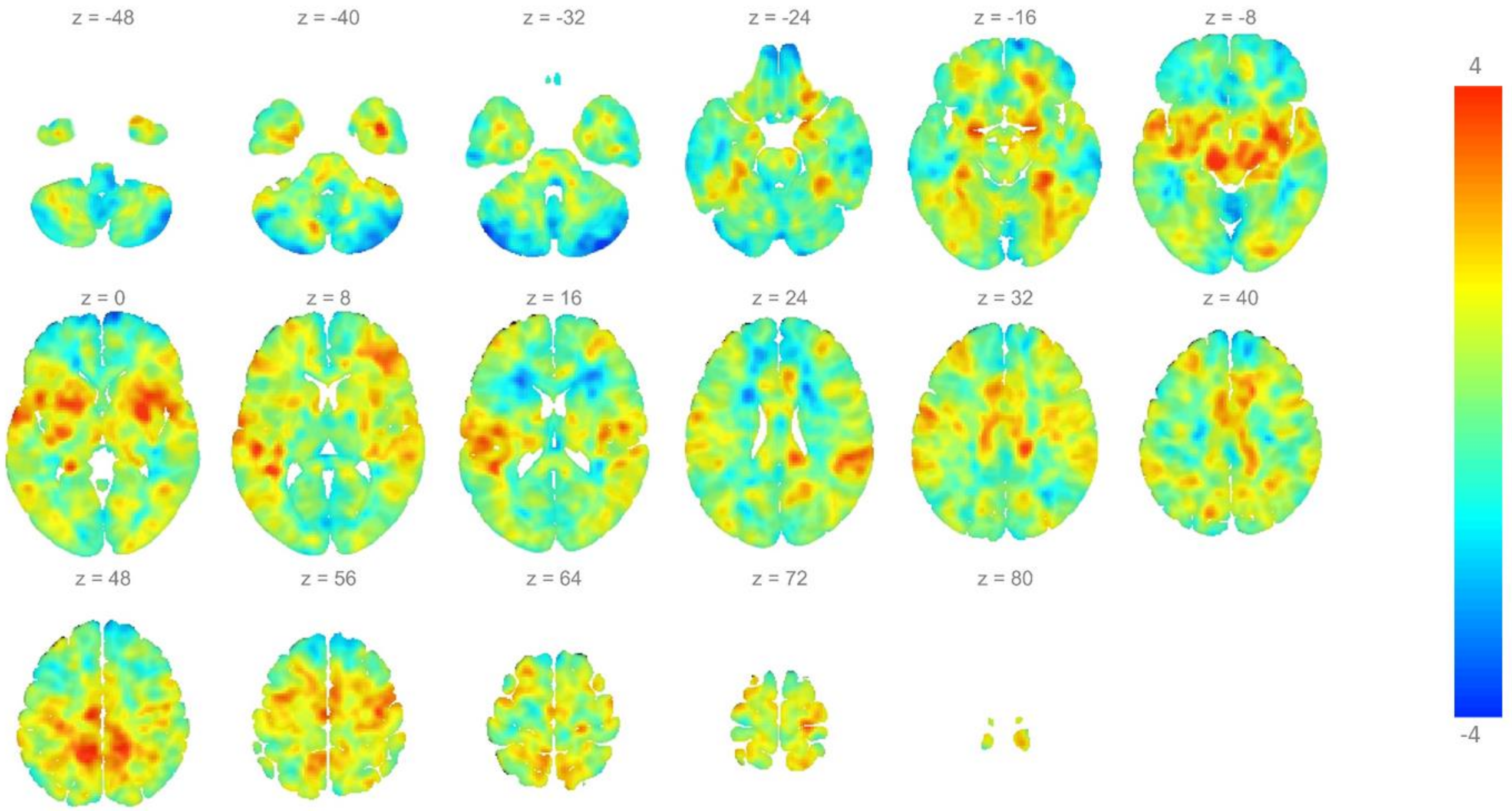

LEFT TEMPORAL PARIETAL JUNCTION (-50, -48, 10)

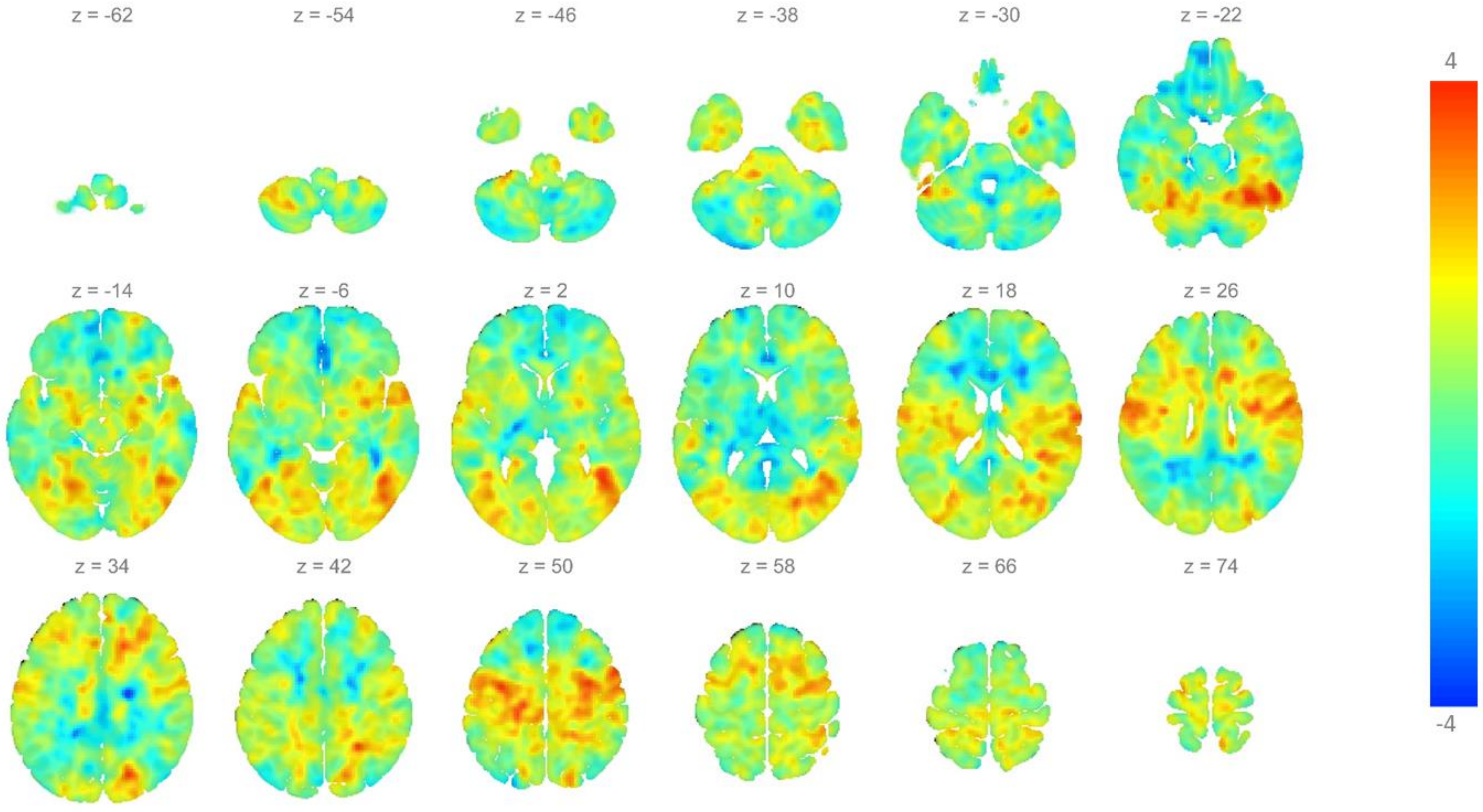

LEFT TEMPORAL PARIETAL JUNCTION (-50, -42, 20)

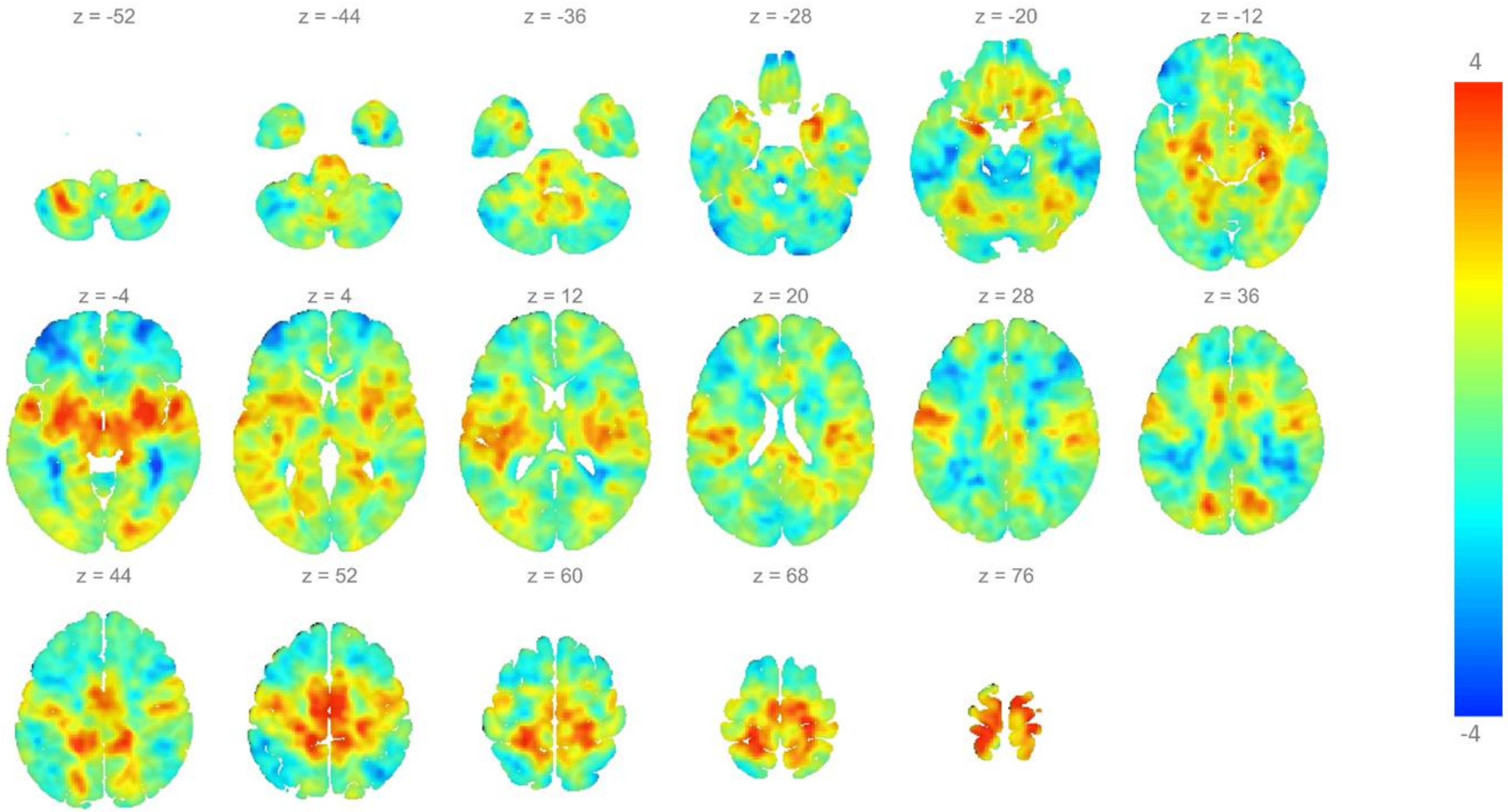

LEFT TEMPORAL PARIETAL JUNCTION (-34, -48, 12)

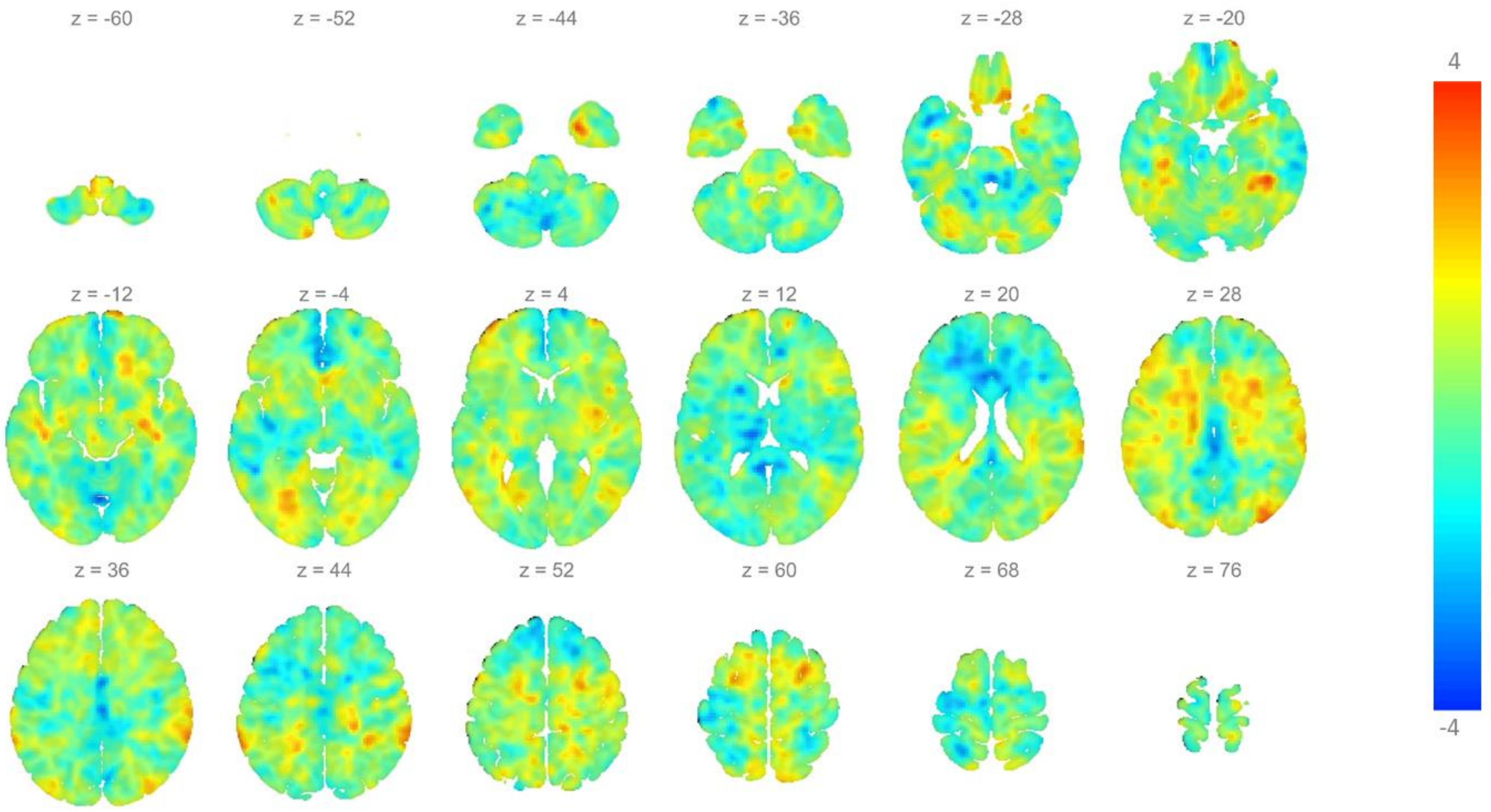

RIGHT TEMPORAL PARIETAL JUNCTION (58, -40, 24)

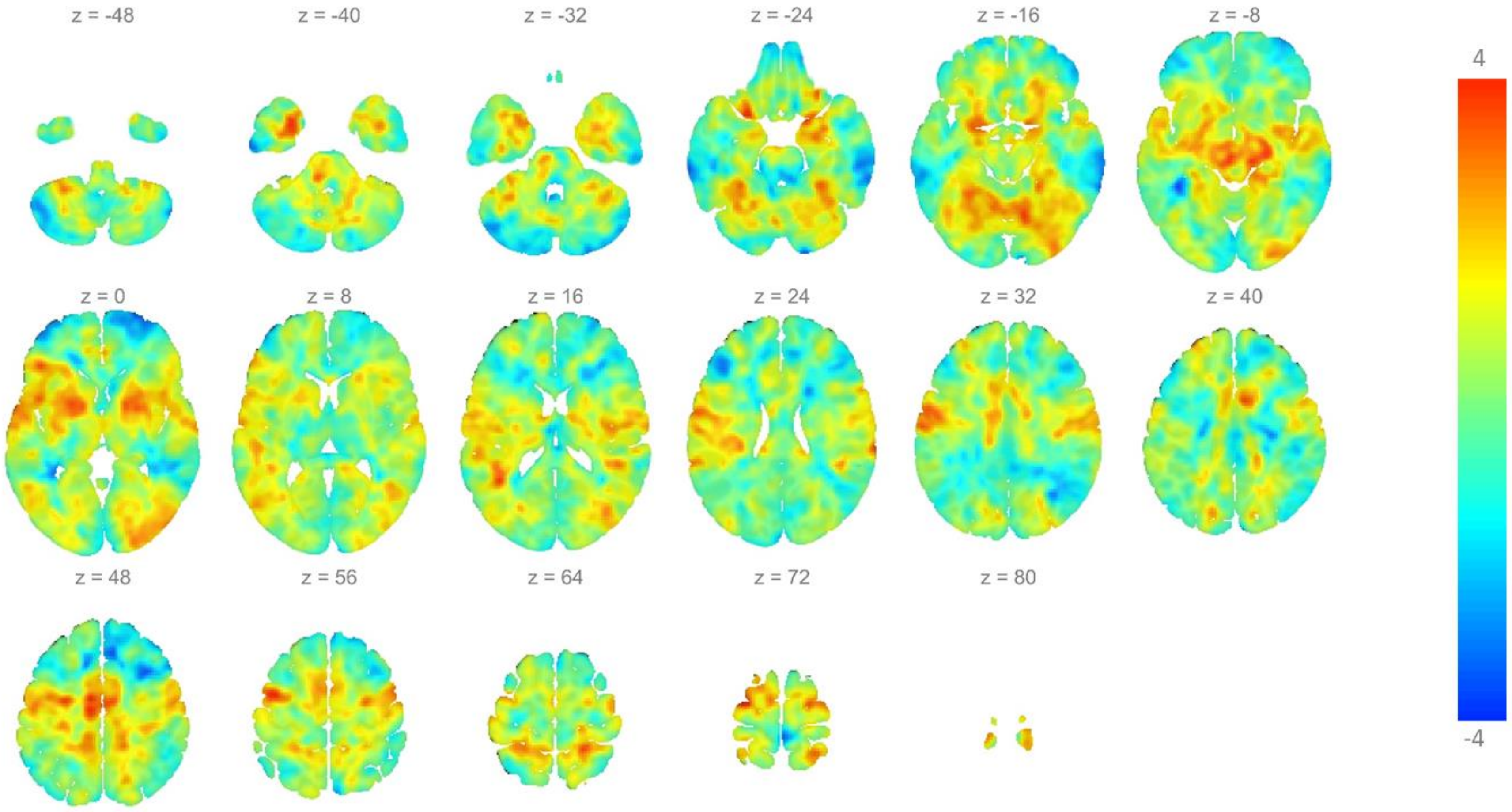

RIGHT TEMPORAL PARIETAL JUNCTION (60, -52, 16)

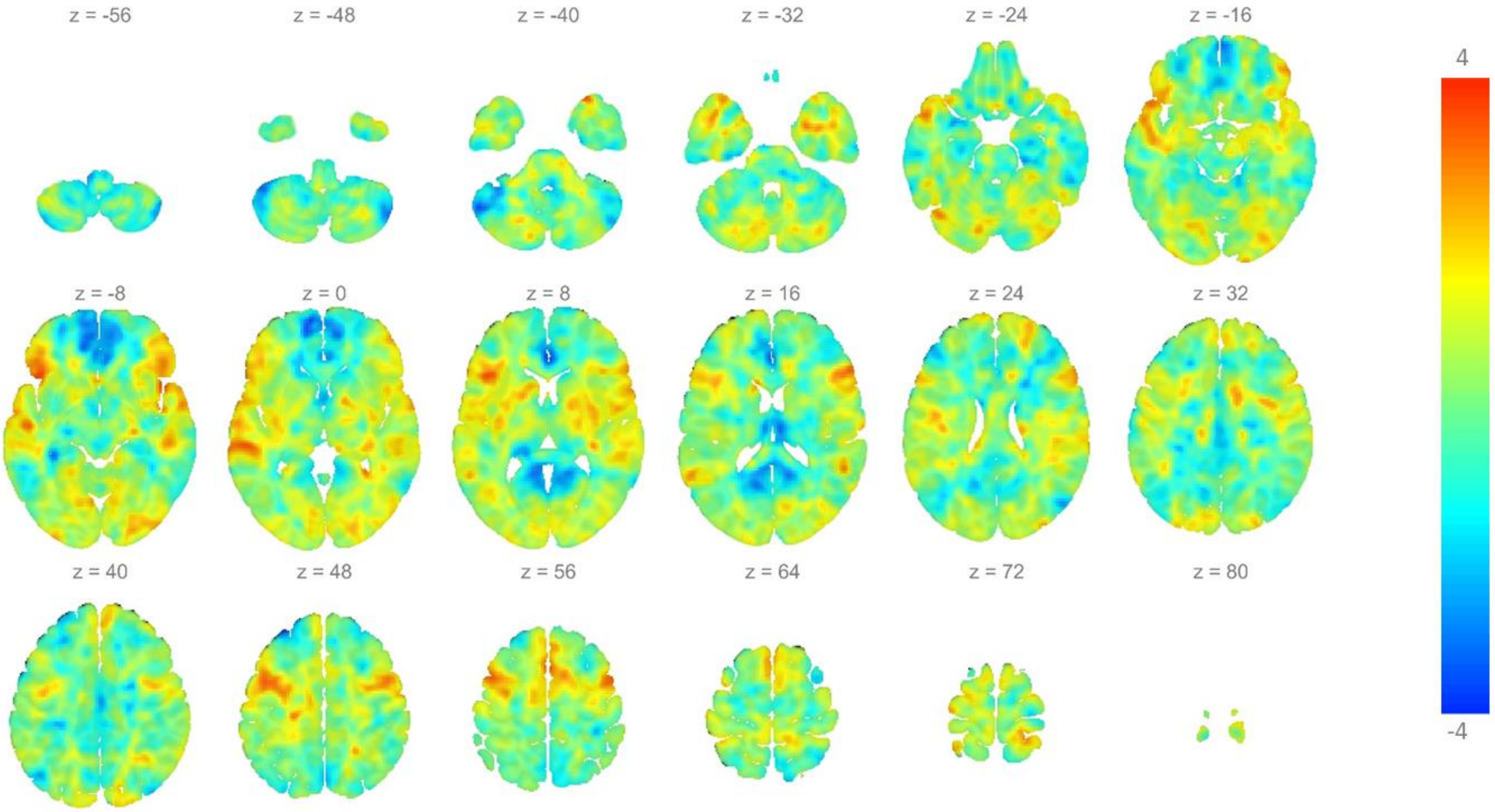

RIGHT TEMPORAL PARIETAL JUNCTION (64, -50, 14)

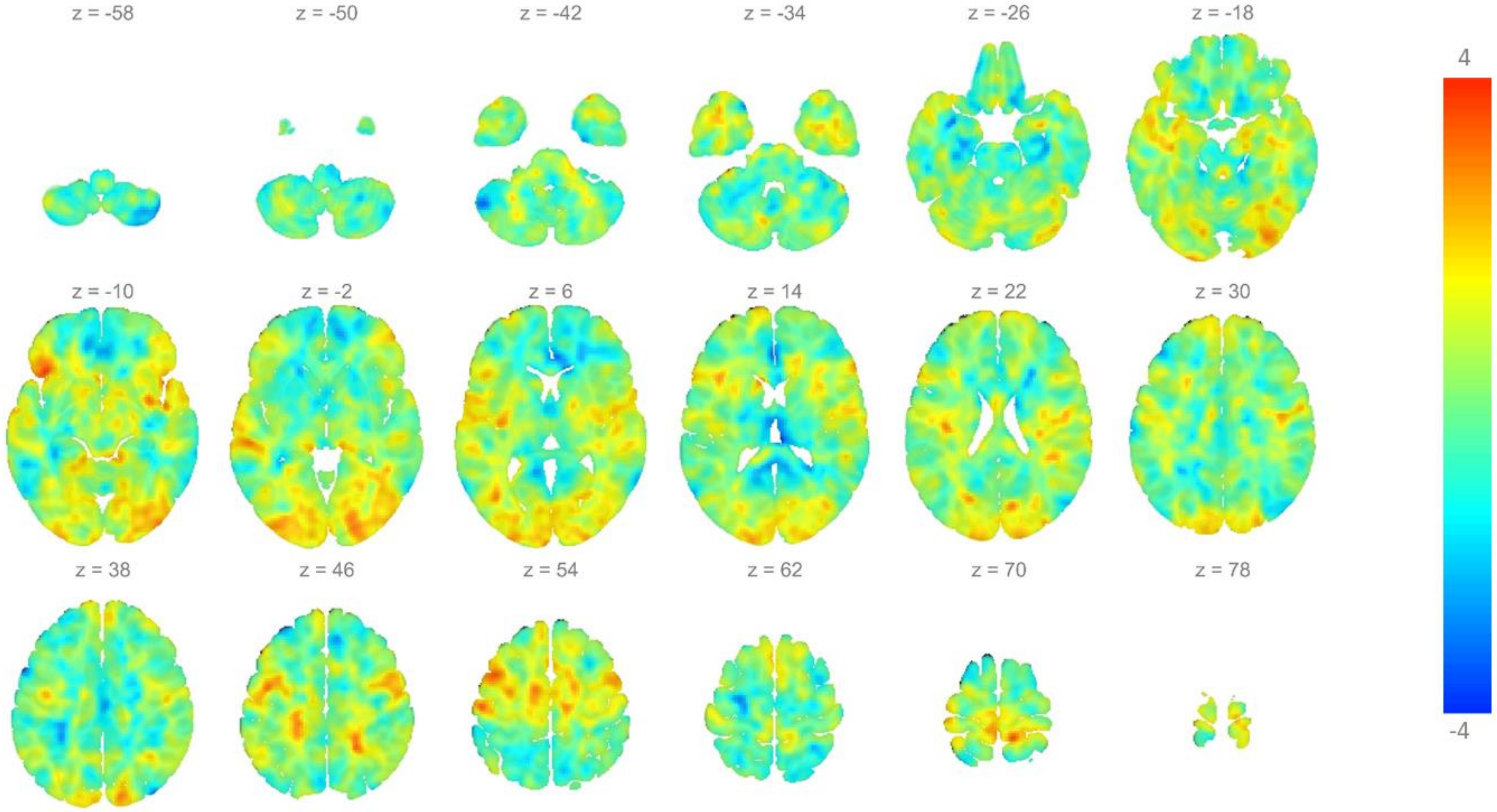

RIGHT TEMPORAL PARIETAL JUNCTION (66, -44, 20)

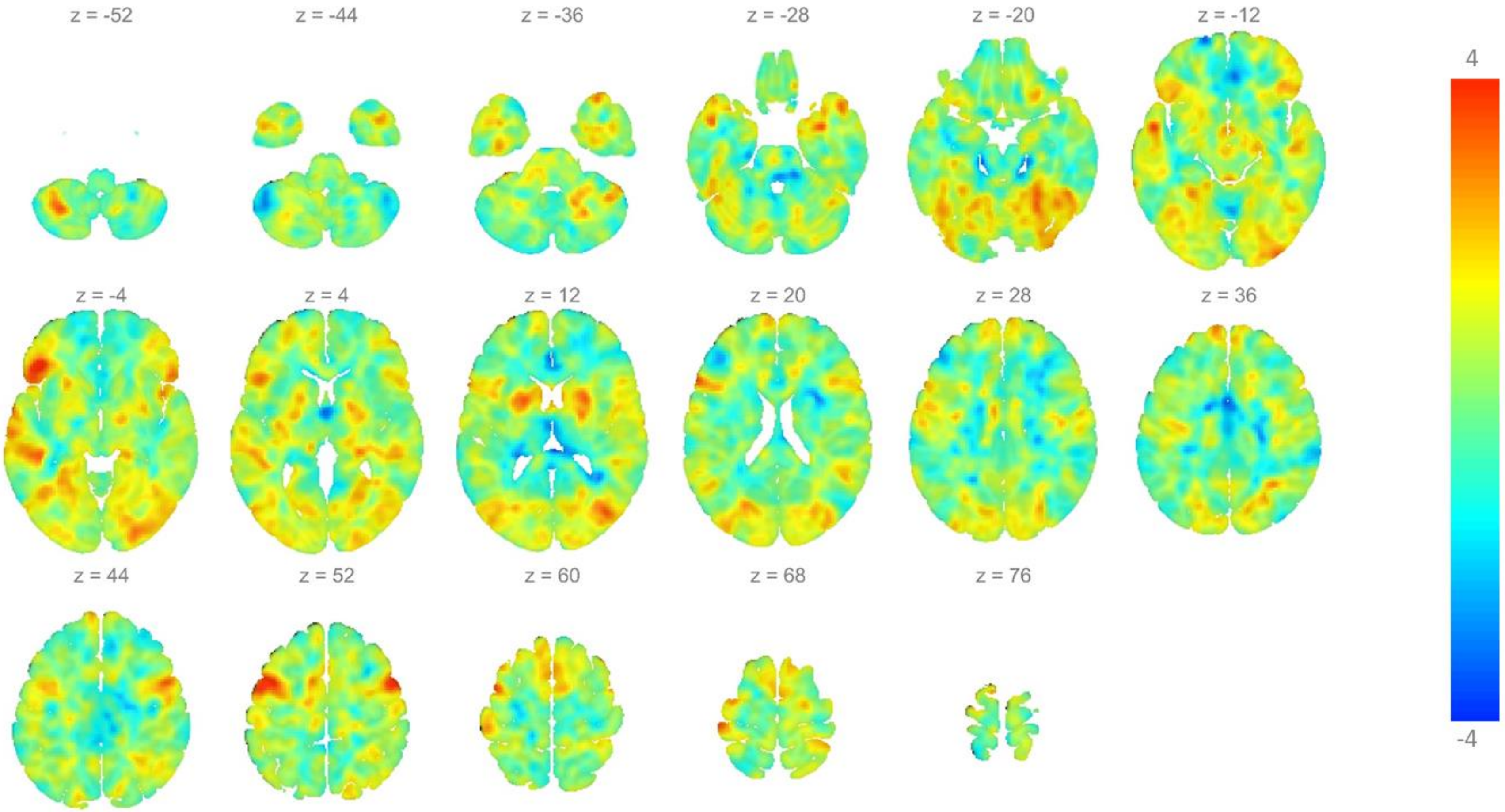

Figure S4. Psychometric curve fitted on the data of all participants taken together (n = 65) from all trials (n = 200) in the visual temporal order judgment (TOJ) task. The function was calculated using the same method as described in the manuscript. The threshold parameter of the function was set to 0.71 since the overall correctness in the adaptive procedure used should have converged to 71%. The lower asymptote was fixed at 0.5 because of the two alternative forced choice (2AFC) form of TOJ task. Data points were pooled in the interval of 2 msec. forming groups with not equal number of elements as shown by different sizes of the blue spots. Dashed line represents extrapolated ranges of the psychometric curve. The threshold of the function unscaled by guessing and lapse rate as defined by Schütt et al. (2016) is indicated by the vertical line as well as its 95% Confidence Interval (CI) is shown. Dotted horizontal lines represent the lower and upper asymptotes.

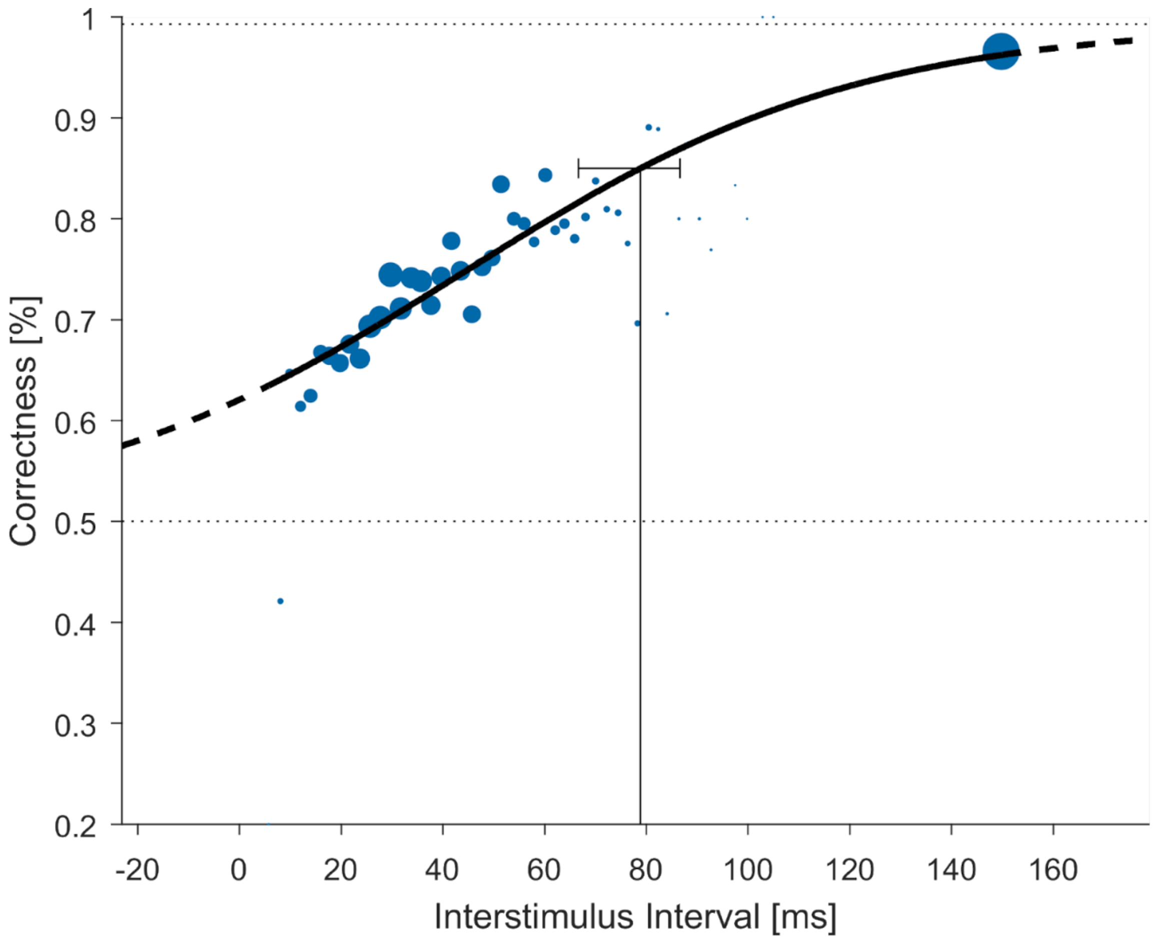

Figure S5. Individual psychometric curves calculated from all trials (n = 200) of the visual TOJ task in each participant. Since the overall correctness in the TOJ task converged to 71%, the threshold parameter of the function was set to 0.71. It is worth noting that the value of threshold of the psychometric is not equal to the temporal order threshold TOT value, defined as the interval for 75% correctness. Dashed line mark extrapolated ranges of the psychometric curve.

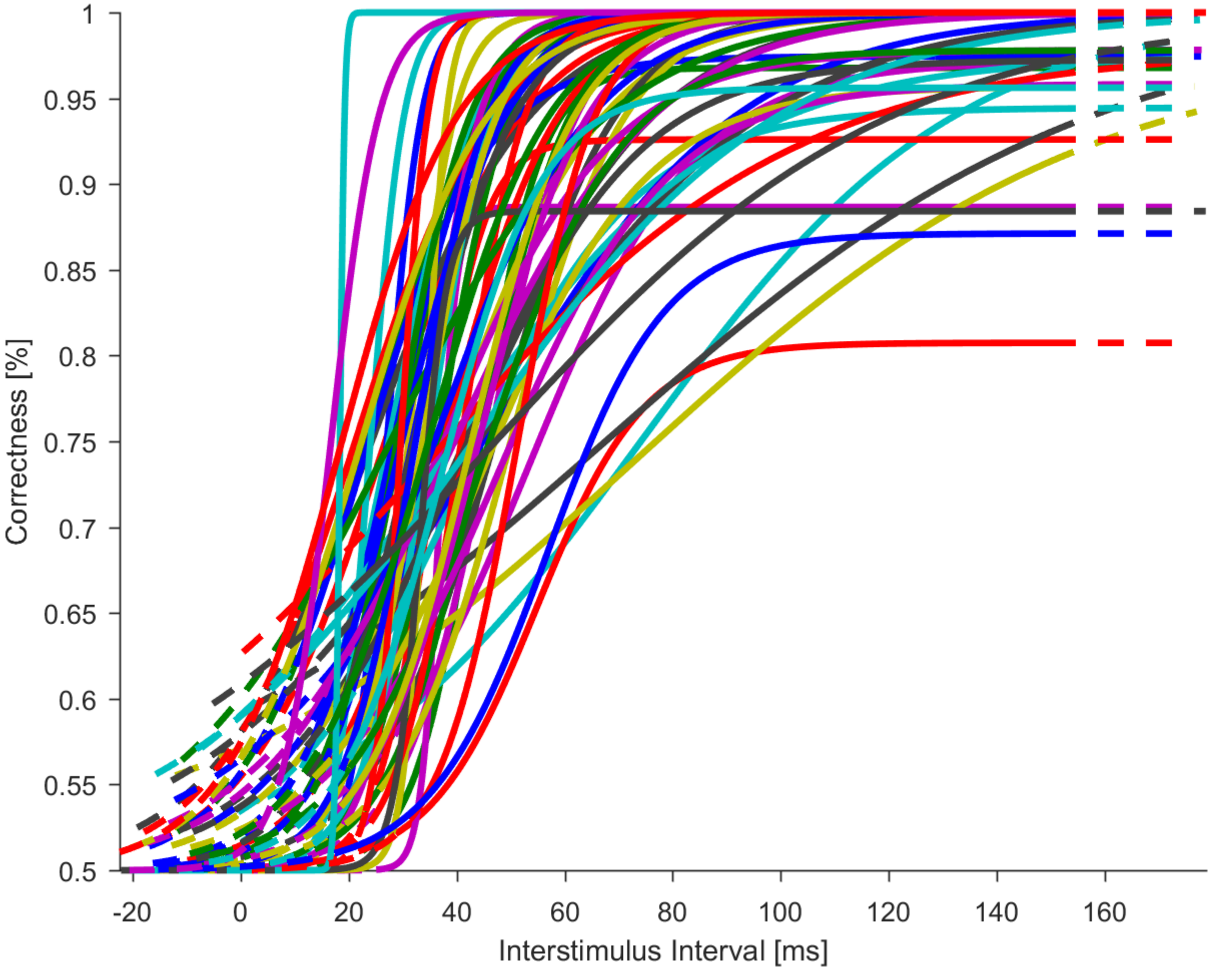

Supplement: Supplementary file 1 — Supplementary Information. [file 41598_2022_20309_MOESM1_ESM.pdf]
